# Supplementary material for: Domain-specific insight into the recognition of BH3-death motifs by the pro-survival Bcl-2 protein
Source: Biophys J. 2022 Nov 2;121(23):4517–25. doi: 10.1016/j.bpj.2022.10.041 (PMC9748362; doi:10.1016/j.bpj.2022.10.041)
Supplement: Document S1. Figures S1–S9 [file mmc1.pdf]

Biophysical Journal, Volume *121*

Supplemental information

Domain-specific insight into the recognition of BH3-death motifs by the pro-survival Bcl-2 protein

Ameeq Ul Mushtaq, Jörgen Ådén, Katan Ali, and Gerhard Gröbner

Supplemental information

Domain specific insight into the recognition of BH3-death motifs by the pro-survival Bcl-2 protein

Ameeq Ul Mushtaq, Jörgen Ådén, Katan Ali, and Gerhard Gröbner

Figure S1: SDS-PAGE of purified, activated Bax protein.

Figure S2: Secondary structure determination of refolded, activated Bax protein.

Figure S3: ITC experiment of binding intact human Bax protein to Bcl-2 protein.

Figure S4: Assignment of Bcl-2 glycine residues.

Figure S5. Glycine specific insight into Bcl-2 domain dynamics.

Figure S6: Titration of mBax-BH3 peptide against Bcl-2 protein.

Figure S7: $^{1}H^{15}N$ peak intensities of Bcl-2 glycines against mBaxBH3 peptide titration.

Figure S8: Glycine residue specific affinities.

Figure S9: Schematic picture of the micelle-embedded Bcl-2 and detergent activated monomeric Bax in DPC micelles.

References

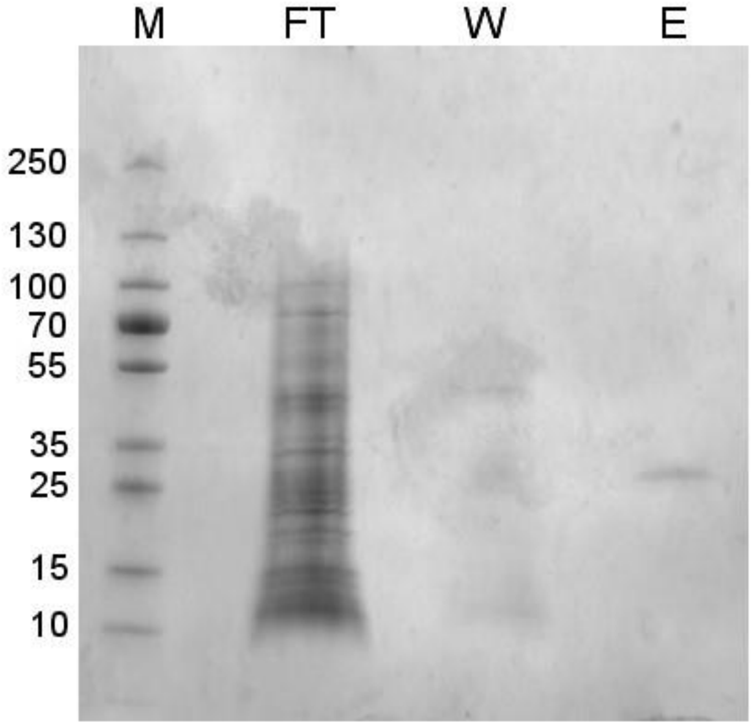

Figure S1. SDS-PAGE of purified, activated Bax protein. Eluted fractions from_nickel affinity purification. Lane M) Molecular weight marker in kDa, FT) Flow-through fraction upon binding refolded, solubilized protein on the column, W) Wash fraction, and E) Eluted, activated Bax protein, recovered from inclusion bodies. Expected molecular weight for Bax with its attached His-tag is 23.3 kDa.

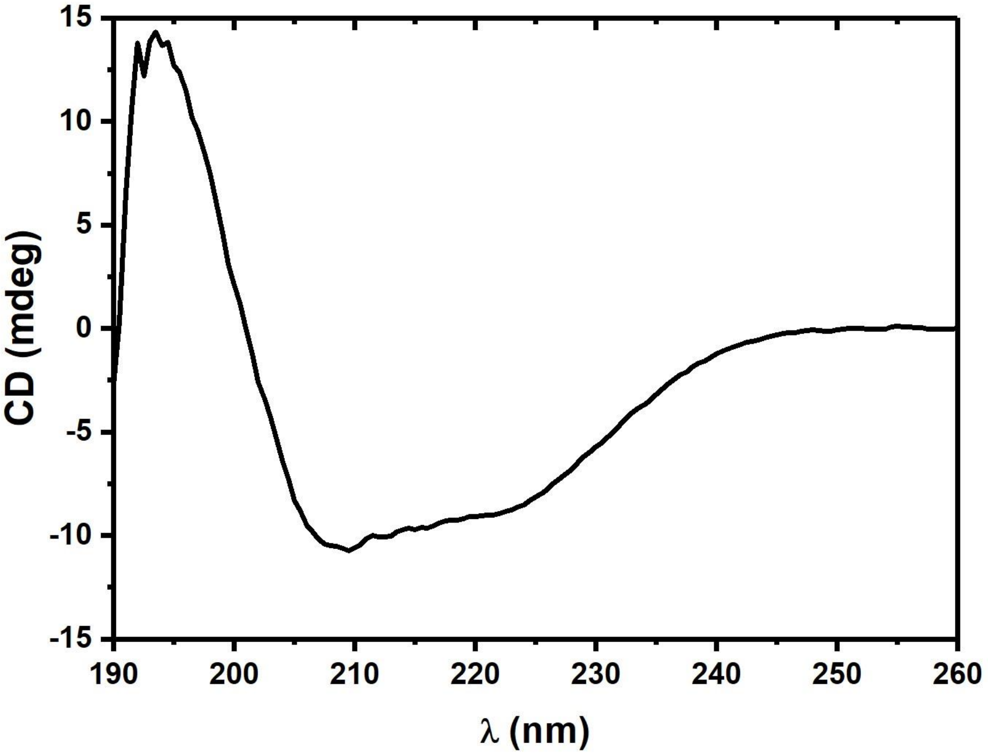

Figure S2. Secondary structure determination of refolded, activated Bax protein. Far-UV CD spectrum of 5 µM activated Bax protein in NMR buffer (20 mM NaPi, 20 mM NaCl, 5 mM DPC, 2 mM TCEP, pH 6.0), recorded at 20 °C. The CD spectrum shows a characteristic helical structure, expected for folded Bax protein.

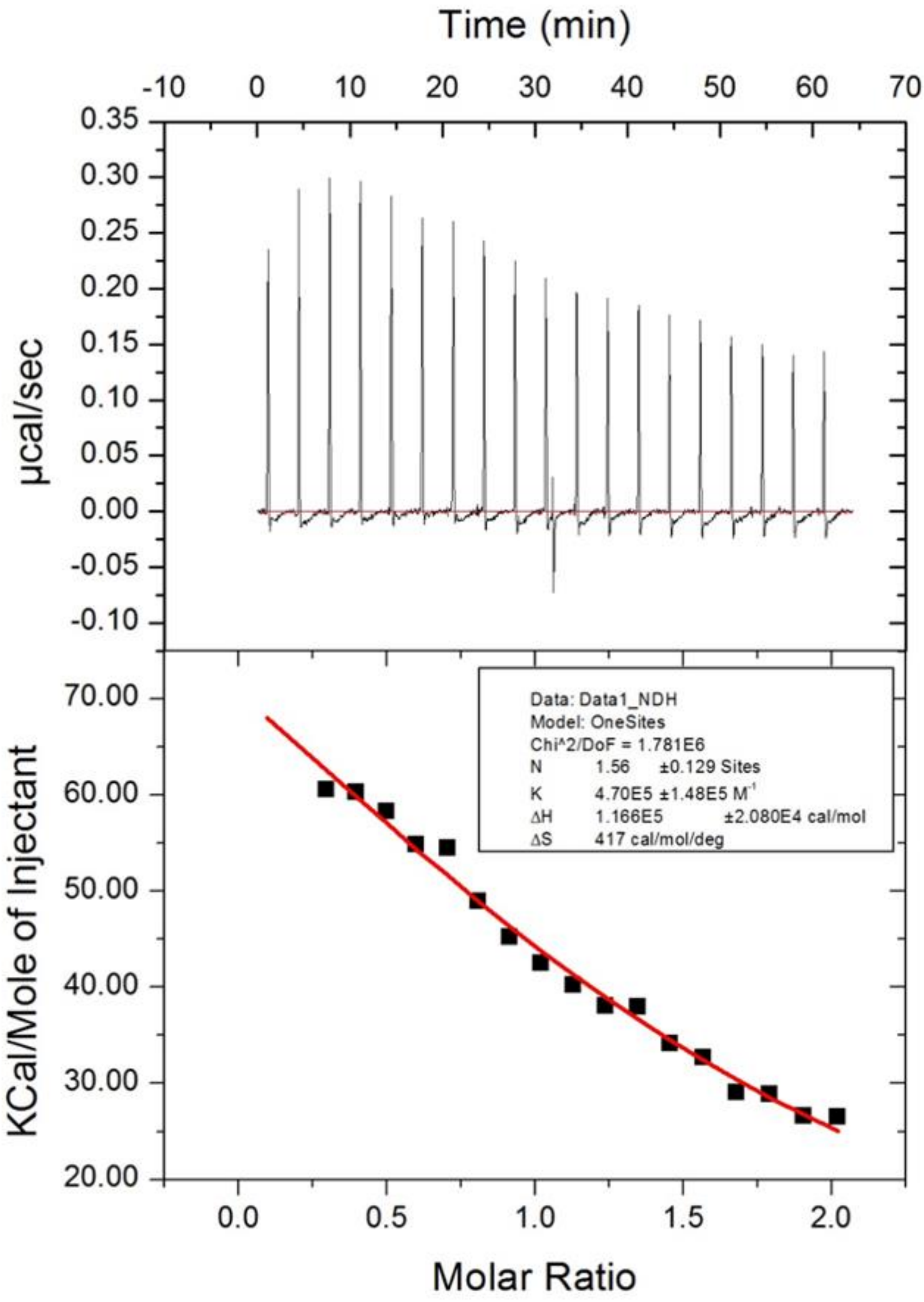

Figure S3. ITC experiment of binding intact human Bax protein to Bcl-2 protein. ITC raw data (top panel) showing differential power (DP) and curve fit (bottom panel) obtained for titrations of 20 µM wild-type human Bax protein into 2 µM intact human Bcl-2 protein. The dissociation constant (K_D) of the binding affinity between both proteins in 5 mM DPC containing NMR buffer was calculated to a K_D value of 2.12 µM. The integrated titration curve (bottom) shows the integrated heat data that was fitted into the one site binding model.

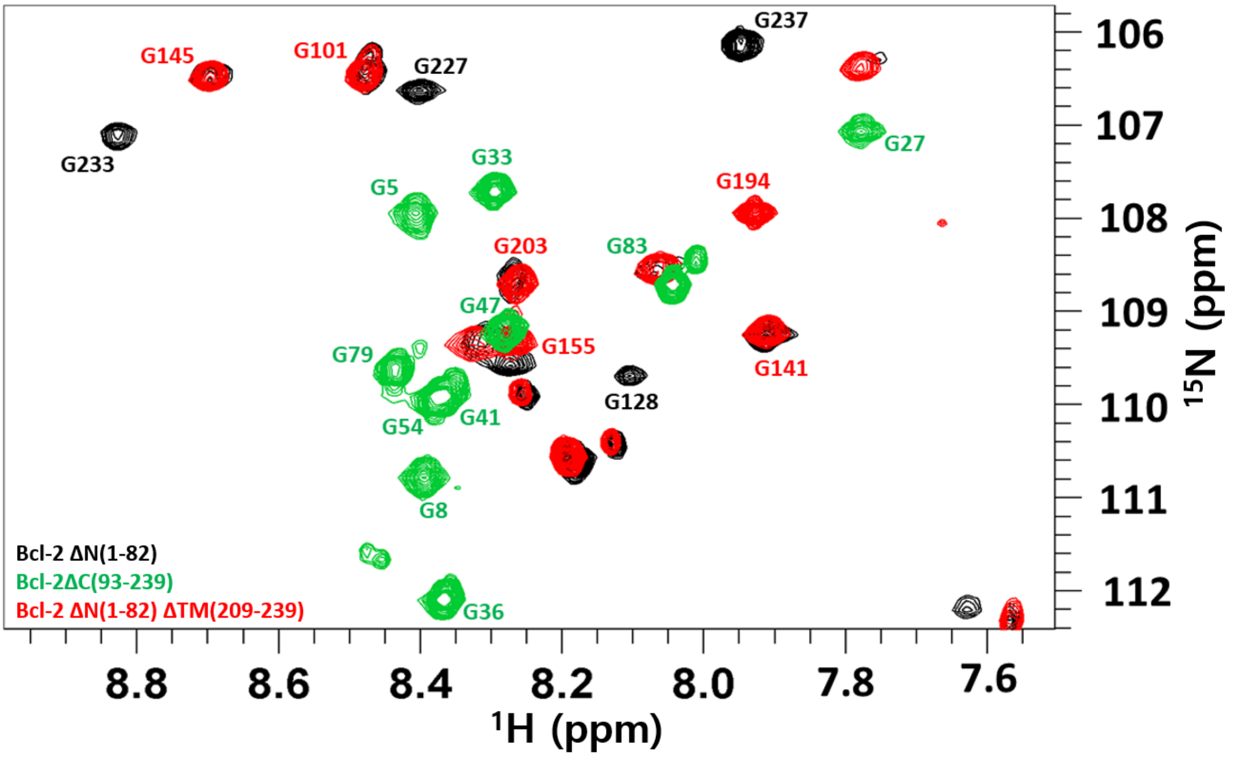

Figure S4. Assignment of Bcl-2 glycine residues. Overlay of the spectral glycine regions of ^{1}H-^{15}N-TROSY-HSQC NMR spectra of Bcl-2 constructs: (black) 0.4 mM ^{15}N-labeled Bcl-2 ΔN(1-82); (green) 0.63 mM ^{15}N-labeled Bcl-2 ΔC(93-239) and (red) 0.25 mM ^{15}N-labeled Bcl-2 ΔN(1-82) ΔC(209-239) protein. All spectra were acquired at 310 K at 850 MHz ^{1}H frequency. Identified glycine residues in the constructs are colored accordingly. Truncation of Bcl-2 protein with fully functional subunits provides less overlapped NMR spectra (s. also [1]).

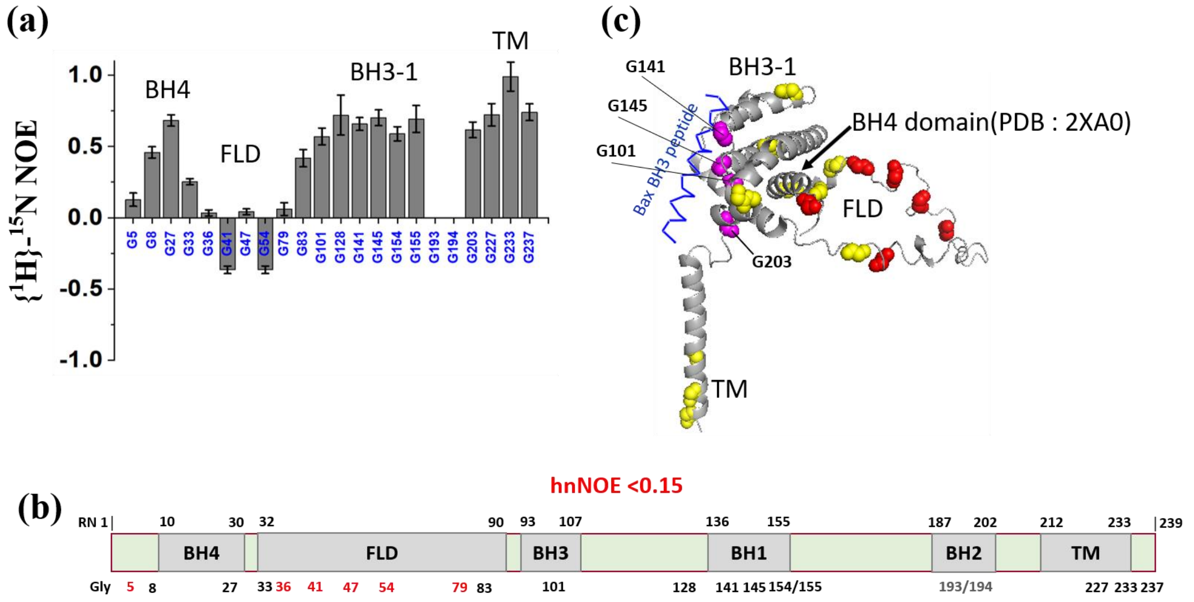

Figure S5. Glycine specific insight into Bcl-2 domain dynamics. (a) Steady-state $\{^1H\}$-^{15}N heteronuclear NOE plot of the glycine amide groups of Bcl-2 showing ps-ns dynamics of domains in a membrane-mimicking micellar environment. ^{15}N-^1H heteronuclear NOE saturation/unsaturation values are plotted on the y-axis between values (−1.0 to 1.0). Errors associated with each NOE value from replicate measurements are shown on top of the bar graphs. **(b)** Glycine residues with hnNOE <0.15 are shown in red along the Bcl-2 sequence. **(c)** Model of the full-length Bcl-2 (where FLD and TM domains are modelled based on the tail-anchored model) derived from truncated Bcl-2 complex (PDB ID: 2XA0). Glycine residues are shown in sphere representations to highlight their distribution in the tail-anchored model, glycines in vicinity to Bax-BH3 peptide are shown in magenta spheres. G141, G145, G101 and G203 are the ones showing major chemical shift perturbations, residues 143-146 i e NWGR of the binding-groove are shown in magenta, glycines' with hnNOE <0.15 are shown in red and the remaining glycines are shown in yellow color spheres.

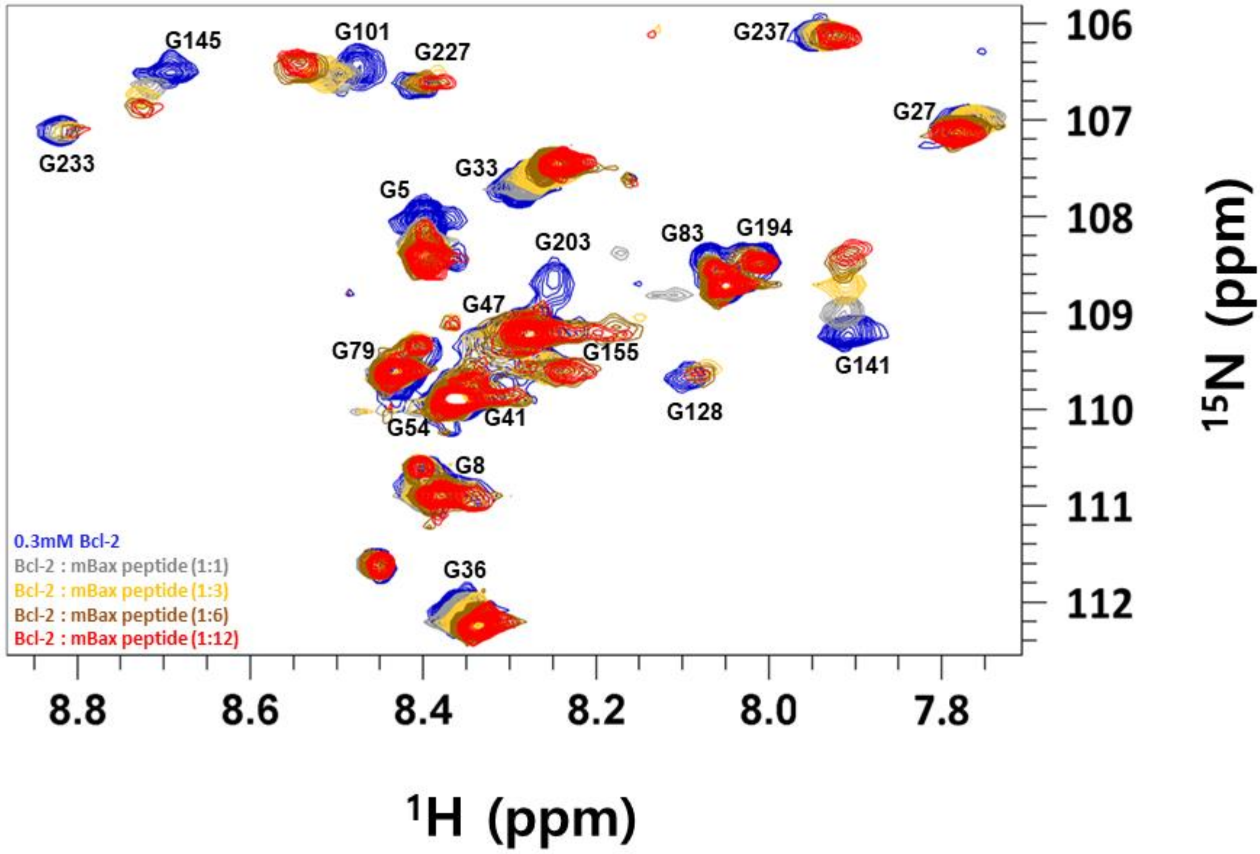

Figure S6. Titration of mBax-BH3 peptide against Bcl-2 protein. Overlay of expanded spectral glycine regions of ^{1}H-^{15}N-TROSY-HSQC NMR spectra showing chemical shift perturbations (CSP's) observed in 0.3 mM ^{15}N-labeled Bcl-2 in NMR buffer upon titration with the Bax-BH3 peptide at various ratios at 310K and at 850 MHz ^{1}H frequency, as described previously [1]. Spectra of Bcl-2 prior (blue) and upon addition of Bax-BH3 peptide added at 1:1, 1:3, 1:6 and 1:12 stoichiometry are indicated in in grey, yellow-orange, brown and red.

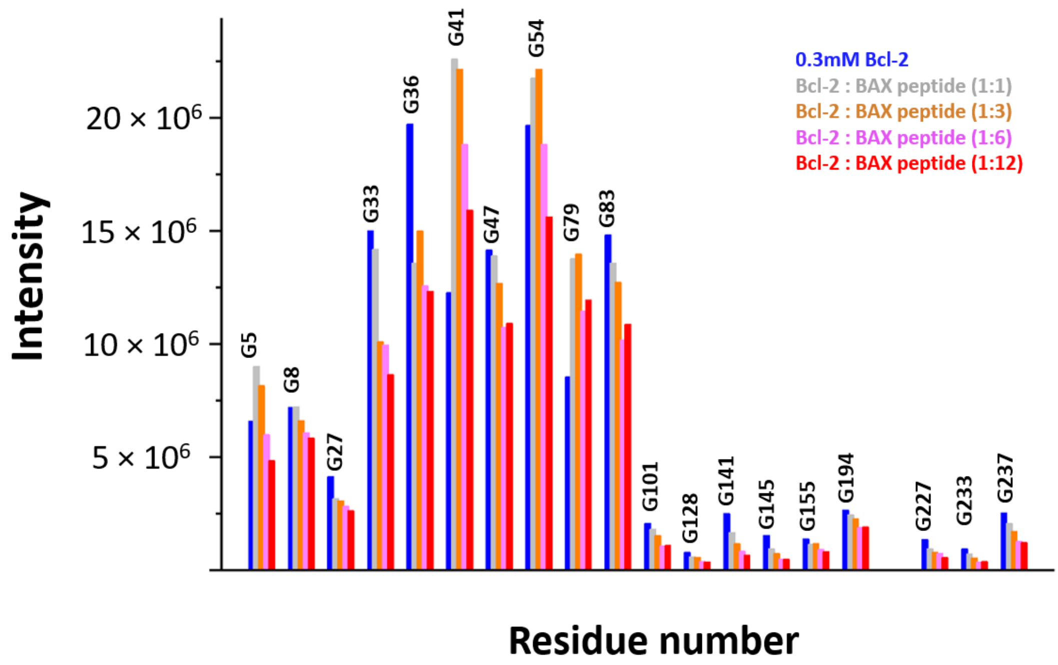

Figure S7. ^{1}H-^{15}N peak intensities of Bcl-2 glycines Bcl-2 as a function of mBax-BH3 peptide concentration. Peak intensities of Bcl-2 glycines against Bax-BH3 peptide at 1:0, 1:1, 1:3, 1:6 and 1:12 protein to peptide molar ratio. Reduced peak intensities of flexible and rigid domains of Bcl-2 along Bax BH3 peptide titration in DPC buffer are plotted.

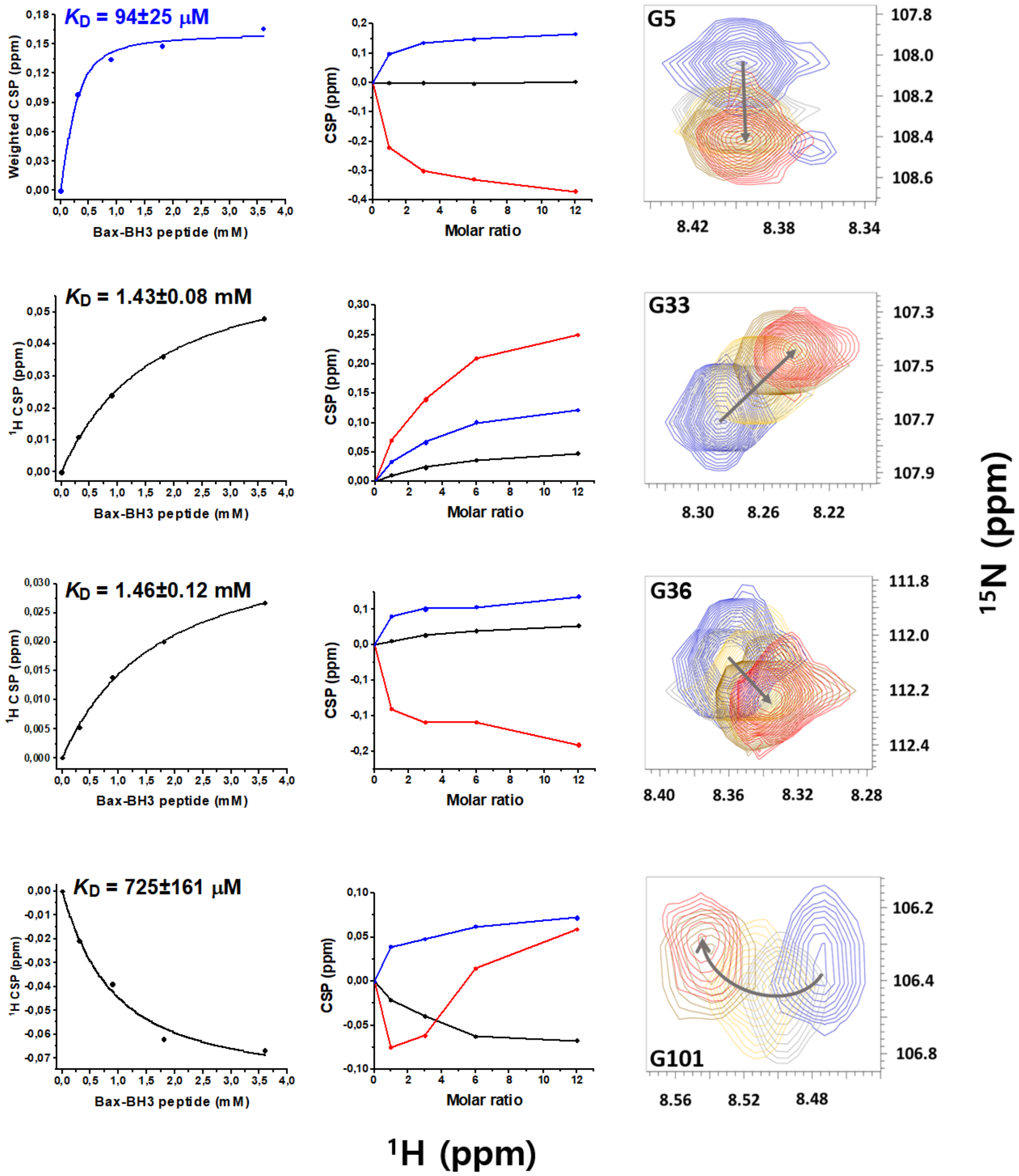

KD = 94±25 μM
Weighted CSP (ppm)
Bax-BH3 peptide (mM)
CSP (ppm)
Molar ratio
G5
KD = 1.43±0.08 mM
1H CSP (ppm)
G33
KD = 1.46±0.12 mM
G36
KD = 725±161 μM
G101
1H (ppm)
15N (ppm)

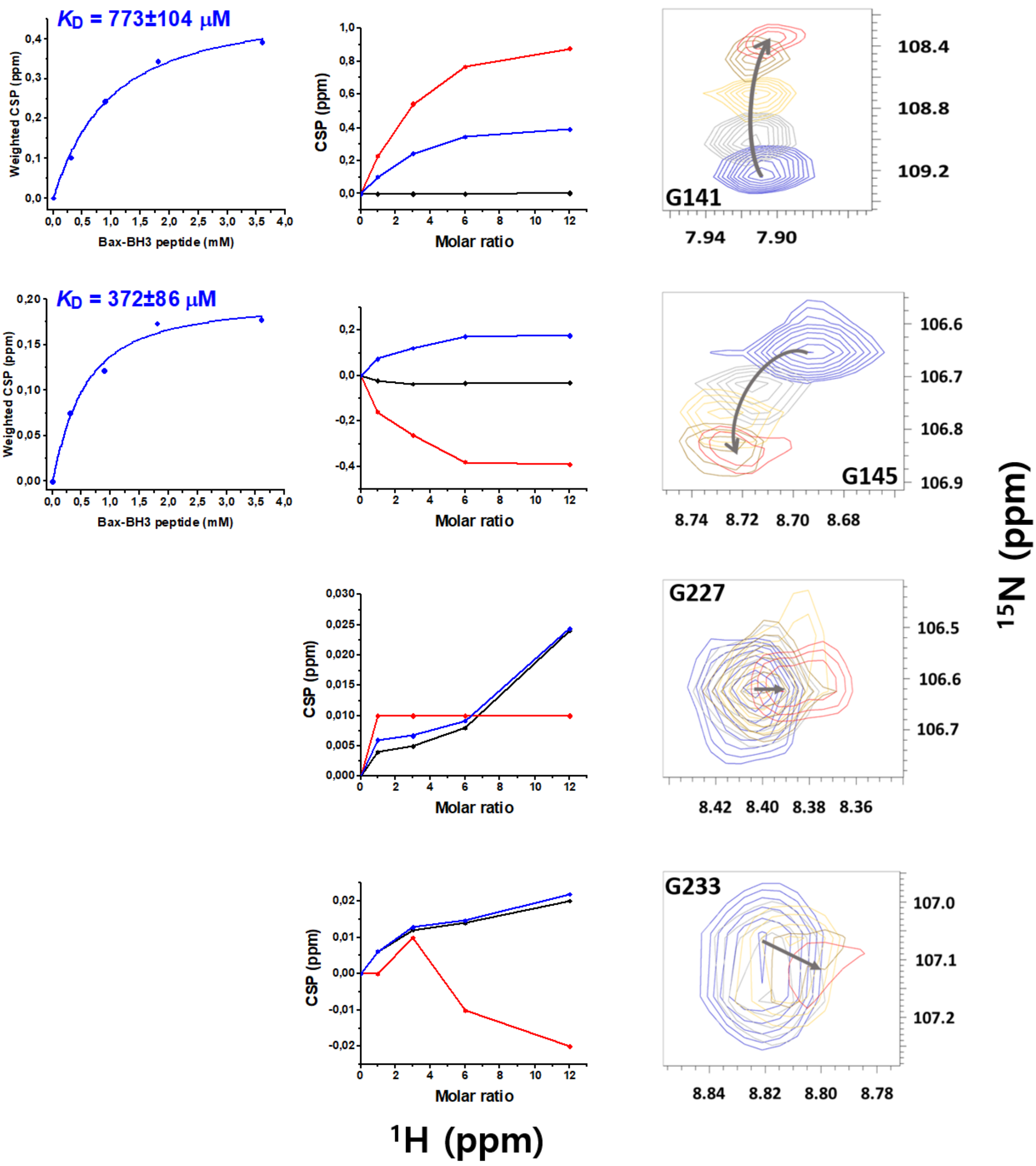

Figure S8. Glycine residue specific affinities. Zoomed boxes (x-axis presents ^{1}H ppm scale; y-axis the ^{15}N ppm scale) show perturbed glycine peaks of the full Bcl-2 spectrum upon addition of the Bax-BH3 peptide. Shown in the middle are the corresponding CSP values for the ^{1}H (black) and ^{15}N (red) nuclei and their weighted average (blue) as described in the Methods section, respectively. For residues showing significant perturbation upon titration (>0.2 ppm), K_D values were calculated and shown (left).

K_D values were calculated either from the ^{1}H, ^{15}N or ^{1}H-^{15}N weighted chemical shifts ($\Delta\delta\ (ppm) = [(\delta H)^2 + 0.1 \times (\delta N)^2)]^{1/2}$) (color coded), based on which fit yielding the lowest chi square value.

K_D values were calculated in OriginPro 2017 (OriginLab Corporation, USA) using the following equation:

$$fb1 = \frac{K_A(E_{tot} + x) + 1}{2 \times K_A \times E_{tot}}$$

$$\sigma = \frac{K_A \times (E_{tot} + x) + 1}{(2 \times K_A \times E_{tot})^2 - x/E_{tot}}$$

$$y = \omega\left(fb1 - \sqrt{(\sigma)}\right)$$

Where E_{tot} is the protein concentration, K_A the association constant, and ω the chemical shift, respectively.

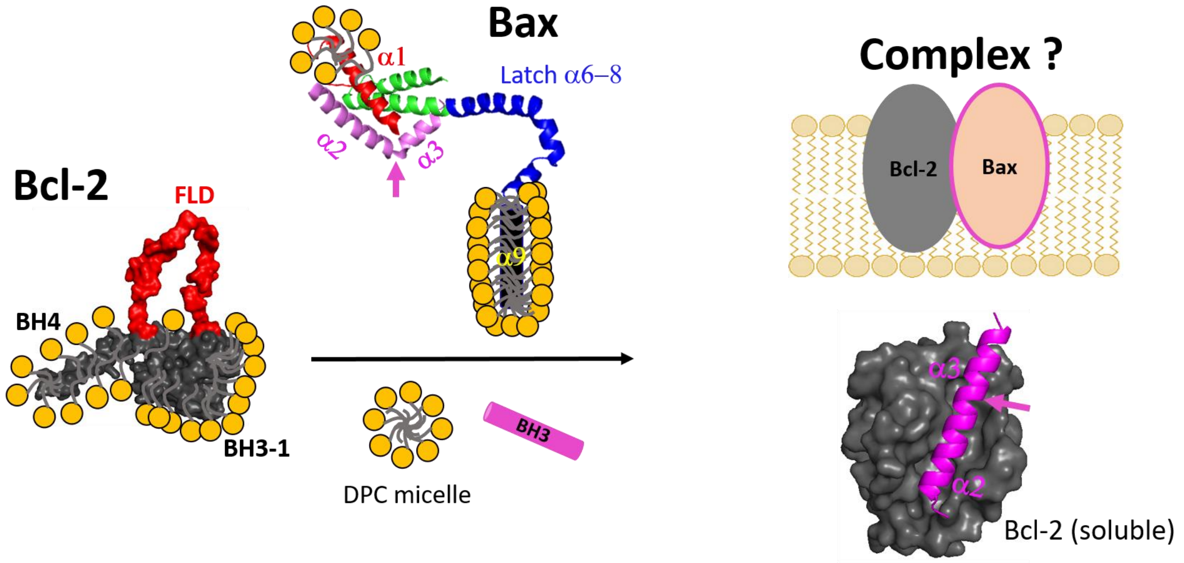

Figure S9. Schematic picture of the micelle-embedded Bcl-2 and detergent activated monomeric Bax in DPC micelles. Detergent-activated monomeric Bax showing exposed BH3 domain (helices 2-3) of the core domain (helices 2-5), latch domain (helices 6-8) and TM (helix 9) domain which is embedded in micelles. Bcl-2 protein in micelles comprises the solvent-accessible regulatory loop domain (FLD), and the micelle embedded BH4 and BH3-1(+TM) domains. Arrows in magenta color show the kink between helices 2 and 3 of activated Bax (PDB ID: 4BD2) which becomes a straight helix in the Bcl-2 bound state (PDB ID: 2XA0).

References

[1] A. Ul Mushtaq, J. Aden, T. Sparrman, M. Hedenstrom, G. Grobner. Insight into Functional Membrane Proteins by Solution NMR: The Human Bcl-2 Protein-A Promising Cancer Drug Target, Molecules 26(5) (2021).
