# Supplementary material for: Domain-specific insight into the recognition of BH3-death motifs by the pro-survival Bcl-2 protein
Source: Biophys J. 2022 Nov 2;121(23):4517–25. doi: 10.1016/j.bpj.2022.10.041 (PMC9748362; doi:10.1016/j.bpj.2022.10.041)
Supplement: Document S2. Article plus Supporting — material [file mmc2.pdf]

Biophysical Journal
Article

Biophysical Society

Domain-specific insight into the recognition of BH3-death motifs by the pro-survival Bcl-2 protein

Ameeq Ul Mushtaq,[1] Jörgen Ådén,[1] Katan Ali,[1] and Gerhard Gröbner[1,*]
[1]Department of Chemistry, University of Umeå, Umeå, Sweden

ABSTRACT Programmed mammalian cell death (apoptosis) is an essential mechanism in life that tightly regulates embryogenesis and removal of dysfunctional cells. In its intrinsic (mitochondrial) pathway, opposing members of the Bcl-2 (B cell lymphoma 2) protein family meet at the mitochondrial outer membrane (MOM) to control its integrity. Any imbalance can cause disorders, with upregulation of the cell-guarding antiapoptotic Bcl-2 protein itself being common in many, often incurable, cancers. Normally, the Bcl-2 protein itself is embedded in the MOM where it sequesters cell-killing apoptotic proteins such as Bax (Bcl-2-associated X protein) that would otherwise perforate the MOM and subsequently cause cell death. However, the molecular basis of Bcl-2's ability to recognize those apoptotic proteins via their common BH3 death motifs remains elusive due to the lack of structural insight. By employing nuclear magnetic resonance on fully functional human Bcl-2 protein in membrane-mimicking micelles, we identified glycine residues across all functional domains of the Bcl-2 protein and could monitor their residue-specific individual response upon the presence of a Bax-derived 36aa long BH3 domain. The observed chemical shift perturbations allowed us to determine the response and individual affinity of each glycine residue and provide an overall picture of the individual roles by which Bcl-2's functional domains engage in recognizing and inhibiting apoptotic proteins via their prominent BH3 motifs. This way, we provide a unique residue- and domain-specific insight into the molecular functioning of Bcl-2 at the membrane level, an insight also opening up for interfering with this cell-protecting mechanism in cancer therapy.

SIGNIFICANCE The mitochondrial programmed cell death (apoptosis) is essential in life and tightly controlled by the Bcl-2 protein family. Its pro-survival member, the Bcl-2 protein itself, resides at the mitochondrial membrane surface where it inhibits cell-killing Bcl-2 members. Unfortunately, its overexpression is a common cause of many cancers. The lack of structural insight into membrane-embedded Bcl-2 prevents a comprehensive understanding of its function. Using NMR on Bcl-2 in a membrane-similar environment, we could identify all glycine residues located in the various functional domains of the protein and use them as reporters to monitor Bcl-2's molecular response upon recognition of BH3-death motifs of apoptotic proteins. These molecular recognition principles will also serve as a promising platform for future cancer drug developments.

INTRODUCTION

Programmed cell death (apoptosis) is an essential process for human development and health (1). In its intrinsic, also called mitochondrial apoptotic pathway, permeabilization of the mitochondrial outer membrane (MOM) and release of apoptotic factors such as cytochrome c from the mitochondrial interior causes final cellular suicide (2–5). To avoid clearance of healthy cells, but to enable removal of harmful and dysfunctional cells, this process is tightly controlled by the Bcl-2 (*B cell CLL/lymphoma-2*) protein family (2,6). Mechanistically, pro- and antiapoptotic Bcl-2 members meet at the MOM where they control membrane permeability and therefore the fate of a cell based on the net outcome of their intricate interactions between themselves, their relative abundancies, as well as their affinities to the membrane environment (5,7–9). Upon apoptotic intracellular stress signals, apoptotic members such as the multidomain Bax (Bcl-2-associated X protein) protein massively recruit to the MOM where they can partially penetrate and oligomerize into MOM-perforating pores that release apoptotic factors and finally cell death (8,10,11). To prevent this fate to healthy cells, the MOM-embedded antiapoptotic Bcl-2 protein can directly sequester any membrane-associated activated Bax to avoid membrane destruction and ensure cellular survival (7,12).

Submitted July 17, 2022, and accepted for publication October 27, 2022.
*Correspondence: gerhard.grobner@umu.se
Editor: Wendy Shaw.
https://doi.org/10.1016/j.bpj.2022.10.041
© 2022 Biophysical Society.
This is an open access article under the CC BY license (http://creativecommons.org/licenses/by/4.0/).

Unfortunately, many tumors escape this natural cell-clearing apoptotic process by overproduction of the cell-protecting Bcl-2 protein, thereby allowing cancerous cells to continue to grow and increase their resistance to therapy, which causes many tumors to be incurable (13,14). Targeting the antiapoptotic Bcl-2 protein is therefore an attractive strategy to either directly induce cell death or to lower the apoptotic threshold to increase the therapeutic effect of other anticancer drugs. However, how the molecular mechanism by which the Bcl-2 protein exerts its cell-protective function by sequestering apoptotic proteins at all, and especially in cancer cells to protect them, is poorly understood due to the absence of any atomic-detail structure for the full-length human Bcl-2 protein itself and its sequestering complexes in a mitochondrial membrane setting.

Recently, we showed that Bcl-2 can be membrane embedded with an exposed flexible regulatory loop domain (12), supporting the hypothesis that Bcl-2 functions at the membrane level. Presumably, Bcl-2 possesses a conformational plasticity that might be essential for its adoption of a membrane-embedded conformation (15,16), where it can inhibit partially penetrated Bax at the membrane level to prevent cell-death-causing pore formation. But how does Bcl-2 sequester Bax at the membrane? Interestingly, multidomain Bcl-2 members with opposite functions, such as Bcl-2 and Bax, share similar secondary structures and contain several conserved Bcl-2 homology (BH) domains (4,5). Formally, they belong to the class of "tail-anchored" membrane proteins due to a C-terminal transmembrane (TM) sequence (16). Despite similar homology, Bax is a soluble protein in the cytosol that can become membrane-associated upon activation (17), whereas Bcl-2 is insoluble and located at the MOM (18). In general, the BH3-BH1-BH2 region in Bcl-2 forms an extended binding groove that recognizes the BH3-death domain motif of Bax and can sequester the entire protein, as studies using soluble Bax and soluble truncated Bcl-2 versions revealed (19,20). Binding of BH3-domains of apoptotic proteins to the binding groove region of Bcl-2 is central for its function. In general, activation and inhibition of apoptotic events occurring at the MOM require protein with BH3 domains to interact with the hydrophobic grooves of other Bcl-2 members (6,21).

Previous structural studies exist only for soluble chimeric and truncated Bcl-2 versions often shortened to 166 aa (22,23), but no structure is available for the insoluble full-length human protein consisting of 239 aa. Although those truncated soluble Bcl-2 variants provided some valuable insight into the function of Bcl-2, those versions do not function by themselves in vivo since they lack key functional features like the long flexible loop domain (FLD) and the hydrophobic C-terminal TM anchor part. Those features make intact human Bcl-2 protein insoluble but membrane active in vivo. No reliable model exists describing the topology of Bcl-2 at the MOM nor the orientation and conformational flexibility of its domains to regulate apoptosis and maintain organismal health. Only recently, we obtained a first direct insight into the location of Bcl-2 as an embedded membrane protein by combined neutron reflectometry and nuclear magnetic resonance (NMR) studies (12). To unravel the molecular basis of the recognition of Bax via its death domain by the Bcl-2 protein and to understand the respective response of the entire Bcl-2 protein and especially its binding groove interface, we here combined liquid-state NMR methods with isotope-labeled, full-length, functional human Bcl-2 protein in a membrane-mimicking micellar environment, a strategy we recently developed (24). By identifying all individual glycine residues distributed across the entire Bcl-2 protein in the corresponding NMR spectra, we were able to trace each glycine residue and protein domain individually to monitor their specific response upon an increasing presence of a Bax-derived 36-mer BH3 domain peptide (residues 49–84 in the Bax sequence (19)). Those concentration- and residue-specific changes were used to determine individual glycine residue-specific affinity constants and to identify which part of the protein that is most effected upon binding and how this event modulates the entire protein organization. Using NMR detectable glycine residues in Bcl-2 as molecular reporters to monitor the domain-specific response of the protein to the binding of ligand provides novel information that can directly be linked to its functional state. This way the study is not only providing new knowledge about fundamental apoptotic regulation, but also about cancer and treatment resistance in a wide perspective, which can serve as a promising platform for drug discovery.

MATERIALS AND METHODS

Bcl-2 protein variants

The Bcl-2 wild-type protein as well as the truncated variants Bcl-2 ΔN(1–82), Bcl-2 ΔTM (208–239), Bcl-2 ΔN (1–82) ΔTM (208–239), and Bcl-2 ΔC(93–239) were expressed in $^{15}NH_4Cl$ or $^{15}NH_4Cl/^{13}C$ glucose-enriched M9 minimal media and purified according to the previously described procedure (24). For Bcl-2/Bax interaction studies, the 36-mer Bax-BH3 peptide (Ac-QPPQDASTKKLSECLRRIGDELDSNMELQRMIADVD-NH_2) from *Mus musculus* (19) was purchased from GenScript (Leiden, the Netherlands) and dissolved as a 20 mM stock using a buffer consisting of 5 mM DPC micelles, 20 mM NaPi, 20 mM NaCl, and 2 mM TCEP at pH 6.0.

Expression and purification of refolded, activated Bax protein

The full-length Bax gene was purchased from GenScript (Leiden, the Netherlands) and subcloned into a pET-15b expression vector (Novagen) (containing a cleavable N-terminal His-tag), which uses the restriction sites NdeI and BamH1, respectively. 1 μL of plasmid was transformed into 100 μL *E. coli* BL21(DE3) competent cells using a standard transformation protocol and plated on agar plates containing 100 μg/mL carbenicillin. The following day, single colonies were picked and used as a preculture, grown in 20 mL 1xLB, supplemented with 100 μg/mL carbenicillin and further incubated at 37°C overnight. Bax protein was

expressed in $^{15}NH_4Cl$-enriched M9 media, prepared as in Mushtaq et al. (12), and grown until $OD_{600} \sim 0.6$; then induced with 1 mM IPTG and further incubated at 37°C overnight. Cells were harvested by centrifugation at 4400x *g* for 30 min and resuspended in 20 mM Tris (pH 7.5) and then stored at −80°C before purification. Purification of Bax was initiated by addition of one tablet Roche Complete Protease Inhibitor (Roche, Switzerland) and 3 μL DNAse I (Invitrogen). Lysis of cells was accomplished by sonication on ice, using a Branson 450 Digital Sonifier (Branson Ultrasonics, USA), and cells were centrifuged at 48,000x *g* for 30 min. The pellet containing Bax in inclusion bodies was resolubilized in 60 mL 20 mM Tris, 0.6% sarkosyl, 200 mM NaCl, 10% glycerol, 7 M urea (pH 8.0), followed by centrifugation at 27,000x *g* for 30 min. The supernatant from this step was dialyzed overnight using a Spectra/Por 12,000–14,000 MWCO membrane (Spectrum Medical Industries, Texas, USA) at 4°C and a buffer consisting of 20 mM Tris, 200 mM NaCl, 10% glycerol, 0.2% Brij-35 (pH 7.8). The dialyzed protein was centrifuged at 27,000x *g* for 30 min and filtered through a 45-μm filter, and it was loaded onto a His GraviTrap column (Cytiva, Sweden), equilibrated with 20 mM Tris, 200 mM NaCl, 10 mM DPC (Dodecylphosphocholine, Glycon Biochemicals, Luckenwalde, Germany), 20 mM imidazole (pH 7.8). After loading the protein to the column, approximately 50 column volumes of this buffer were used for washing, exchanging the detergent into DPC. Bax protein was eluted with 10 mL of 20 mM Tris, 200 mM NaCl, 10 mM DPC, 500 mM imidazole (pH 7.8). Presence of pure Bax (MW = 23.3 kDa with His-tag) could be identified using a 4%–20% SDS gel (Fig. S1). The concentration of Bax was determined using the absorption at 280 nm as 35,980 M^{-1} cm^{-1}. Pure Bax protein was buffer exchanged into 20 mM NaPi, 20 mM NaCl, 5 mM DPC, 2 mM TCEP (pH 6.0) and used for isothermal titration calorimetry (ITC) Bcl-2 binding studies and far-UV CD spectroscopy. The secondary structure of refolded Bax was verified using far-UV CD spectroscopy (Fig. S2). Bax was confirmed to be in its activated state in micelles as previously reported (25) and verified by ITC (Fig. S3).

In vitro Bax BH3 domain peptide binding assay

The Bax-BH3 domain-derived 36-mer peptide (Ac-QPPQDASTKK LSECLRRIGDELDSNMELQRMIADVD-NH2) from *Mus musculus* was ordered from GenScript (Leiden, the Netherlands) and dissolved in NMR buffer (5 mM DPC micelles, 20 mM NaPi, 20 mM NaCl, 2 mM TCEP at pH 6.0). Titration was performed using 0.3 mM ^{15}N-labeled Bcl-2 protein by adding BH3 peptide at 1:0, 1:1, 1:3, 1:6, and 1:12 molar ratios, and ^{1}H-^{15}N-TROSY-HSQC spectra were obtained first for the Bcl-2 protein only, followed by acquiring spectra of the remaining titration points. All NMR spectra for Bcl-2 were acquired at 310 K on an 850-MHz spectrometer (Bruker, Karlsruhe, Germany).

NMR spectroscopy on Bcl-2 proteins

^{1}H-^{15}N-TROSY (transverse relaxation optimized spectroscopy) experiments were performed using 0.3 mM ^{15}N-labeled full-length Bcl-2 protein in NMR buffer (5 mM DPC micelles, 20 mM NaPi, 20 mM NaCl, 2 mM TCEP at pH 6.0). All 2D ^{1}H-^{15}N TROSY experiments were performed using 16 scans, time domain sizes of 256 (^{15}N) × 2048 (^{1}H) complex points, and sweep widths of 11,029.412 Hz and 2412.313 Hz along the ^{1}H and ^{15}N dimensions, respectively. Steady-state ^{15}N-{^{1}H} heteronuclear NOE spectra were measured with either 5-s delays between each free induction decay or 2-s delays followed by 3-s-long series of 120° nonselective ^{1}H pulses, as described in Zhu et al. (26). Interleaved ^{15}N-^{1}H NOE experiments were acquired with time domain size of 256 × 2048 complex points, sweep width 10,204.082 Hz and 2411.963 Hz along ^{1}H and ^{15}N dimensions, respectively, with 16 scans at 318 K. NMR experiments were performed at 850 MHz using a Bruker Avance III spectrometer equipped with a triple-resonance TCI cryoprobe. The pulse programs were obtained from the Bruker TopSpin 3.6.2 library and data were processed and visualized using TopSpin 3.6 (Bruker Biospin, Germany).

Isothermal titration calorimetry

ITC experiments were performed using a MicroCal iTC200 (Malvern instruments). Each experiment consisted of 19 injections, using injection volumes of 2 μL, and injection times of 5 s, and a 150-s delay between each injection. For all the experiments, a stirring speed of 1000 rpm was used. 1 or 2 μM Bcl-2 protein concentrations were used in the sample cell, and 20 μM Bax was used in the syringe. All measurements were carried out at 25°C in ITC buffer (5 mM DPC micelles, 20 mM NaPi, 20 mM NaCl, 2 mM TCEP at pH 6.0). The raw data were integrated and analyzed using single binding site models provided in the MicroCal-enabled Origin software (OriginLabs).

RESULTS AND DISCUSSION

To unravel the molecular recognition and inhibition process between opposing members of the Bcl-2 protein family, we have chosen two prominent members, namely the antiapoptotic Bcl-2 protein itself and its counterplayer, the apoptotic Bax with its universal BH3-death motif enforcing its tight and preferential interaction with the Bcl-2 protein (19). By using solution-state NMR on ^{13}C and/or ^{15}N isotope-labeled variants of intact human Bcl-2 protein, we could identify its individual glycine residues and follow their response individually upon addition of a mouse-derived Bax-BH3 motif, presented by a 36-mer peptide (aa 49–84 in the Bax sequence). Their response is visible in perturbations of their individual NMR chemical shifts (27). Since those shifts are sensitive to changes in the local conformation and environment, they are ideal to characterize ligand binding and thereby the individual functional domains of the Bcl-2 protein. This way we could identify which residues and domains are involved in the recognition and binding of BH3-death motifs. Based on the individual glycine affinities derived by analysis of the titration-dependent chemical shift perturbations (CSPs) in the glycine-specific NMR signals, we developed a model with residue-specific resolution.

Identification of glycine residues as individual probes across the entire Bcl-2 protein

NMR experiments on fully functional human Bcl-2 protein in membrane-mimicking DPC-based micelles provided approximately 190 residue peaks, as seen in the ^{1}H-^{15}N-TROSY-HSQC NMR spectrum in Fig. 1 *a*. By combining a range of triple-resonance NMR assignment experiments and further assignment strategies based on truncated Bcl-2 constructs (see Fig. S4), we were able to identify all glycine residues, as visible in the insert in Fig. 1 *a* and expanded in Fig. 1 *b*. Glycines distributed along the Bcl-2 sequence can be used as markers for the different functional domains (FLD, BH4-BH1, TM), as shown in Fig. 1 *c*. Backbone amide protons of glycines from the flexible loop regions

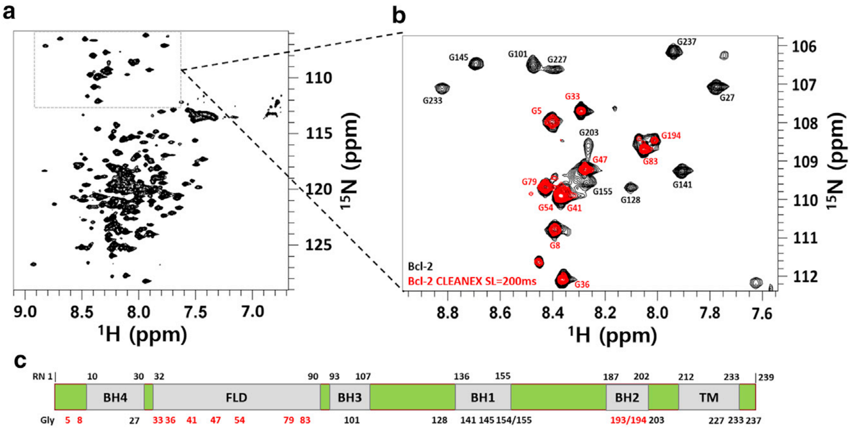

FIGURE 1 Identification of glycine residues in the full-length Bcl-2 protein by NMR. (*a*) ^{1}H-^{15}N-TROSY-HSQC NMR spectrum of 0.3 mM ^{15}N-labeled Bcl-2 in NMR buffer (5 mM DPC micelles, 20 mM NaPi, 20 mM NaCl, 2 mM TCEP at pH 6.0). Dashed box on the top shows the spectral glycine region. (*b*) Overlay of the zoomed spectral glycine region with assigned glycine residues (as indicated) with the corresponding spectral region based on CLEANEX ^{1}H-^{15}N-TROSY-HSQC experiments using 200 ms spin-lock (in red) under identical sample conditions. All spectra were acquired at 310 K and at 850 MHz ^{1}H frequency. (*c*) Schematic map of the Bcl-2 sequence showing BH4, FLD, BH3, BH1, BH2, and TM domains and glycine residues being present in these domains (labeled in red and solvent accessible) and black (solvent inaccessible). To see this figure in color, go online.

are solvent exposed (red color), whereas other glycines (black) are buried or involved in the Bcl-2/micelle system and are not exposed to the solvent, as shown in Fig. 1 *b* using chemical exchange NMR spectroscopy (CLEANEX). The conclusion that the micellar environment faithfully mimics the native membrane environment of Bcl-2 is further supported by steady-state ^{15}N-{^{1}H} heteronuclear NOE plots of the glycine amide groups of Bcl-2 (Fig. S5), showing ps-ns dynamics of domains as expected for Bcl-2 residing in a membrane setting (12,15).

As derived from Figs. 1 and S5, most prominent are the seven solvent-exposed and highly flexible glycines belonging to the natively disordered loop region that links the Bcl-2 homology BH4 and BH3 domains. This regulatory FLD (aa 34–90) stretches into the aqueous cellular interior where it can sense cytosol signals and become phosphorylated at multiple sites for controlling the activity of Bcl-2 (12,28,29). The FLD glycines at both ends (G33 and G83) are slightly more ordered (positive heteronuclear NOE values) presumably due to the proximity to the highly structured BH4 and BH3 domains.

Other exposed glycines are found at the N-terminal part before the BH4 domain with G5 being flexible and exposed, and also G193/G194 belonging to the BH2 domain where they link the short α7, α8 amphipathic helices together, as seen in Figs. 1 *b* and S5 *a*. All those glycines are located at the membrane surface, facing the cytosol (12). All other glycines are part of the membrane-buried domains and therefore not solvent accessible, as seen in Fig. 1 *b*; and they are also dynamically restricted as confirmed by our ^{15}N-{^{1}H} heteronuclear NOE experiments (Fig. S5 *a*), which agree with the general organization of Bcl-2 embedded in a membrane environment (12,15,16).

Comparison with previous solid-state NMR and neutron reflectometry studies on Bcl-2 embedded in lipid bilayers (12) and refolding experiments into detergent systems using a soluble Bcl-2 variant without its TM domain (24) clearly shows that the micellar environment used here reflects well the Bcl-2's globular fold into a compact membrane-like state. This is also visible by most residues buried and motionally restricted in a micellar environment, whereas the FLD region is flexible and solvent exposed, as seen here in Figs. 1 and S5; this is an observation in good agreement with the membrane-embedded case (12) and other Bcl-2 relatives like the soluble Bcl-x$_{L}$ and Bcl-w, which adopted similar detergent-induced structures for functioning in membranes (30,31). Taken together, it shows that our arrangement of full-length Bcl-2 well presents its domain topology in vivo of being surrounded by the MOM systems, something which soluble truncated Bcl-2 versions lack. The glycine residues with their distribution across the entire Bcl-2 protein turn out to be ideal reporters for monitoring changes in the entire protein as well as local changes occurring upon binding of apoptotic proteins via their BH3 motif.

Perturbations in the Bcl-2 protein upon binding of the Bax-BH3 motif

Upon addition of Bax-derived BH3 motif to the Bcl-2 protein, we could monitor the glycine residue-specific response as concentration-dependent CSPs in the corresponding NMR spectra, as seen in Fig. S6. The CSP deviations between free and bound state and their positions in the Bcl-2 protein are visualized in Figs. 2 *a* and *b* at 1:12 stoichiometry of Bcl-2 to Bax-BH3 motif. Already, the weighted chemical shift differences for ^{1}H and ^{15}N show major changes in the regions of Bcl-2 associated with binding apoptotic proteins. Inspecting those changes in detail for both nuclei in Fig. 2 *b* enabled us to identify the Bcl-2 domains involved in the binding of Bax-BH3 motif by exploiting the backbone glycine CSP variations.

The binding groove forming BH3-BH1-BH3 domains (G101 to G203) displays major contributions to the binding, as seen in Fig. 2, and also in ^{1}H-^{15}N peak intensity changes (Fig. S7). This is not surprising, since the four subpockets in this region drive the binding to hydrophobic residues located

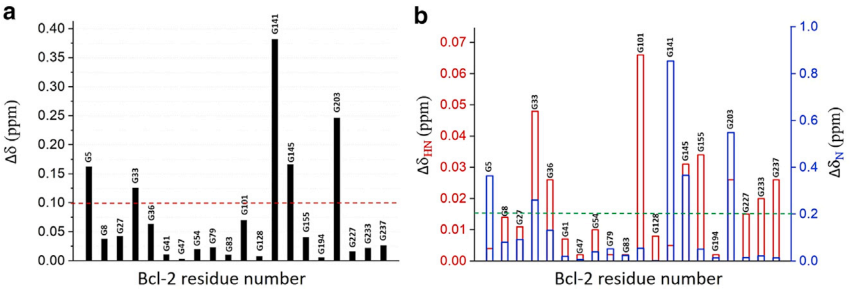

FIGURE 2 Chemical shift response of glycine residues for identification of Bcl-2 domains involved in binding: (*a*) Chemical shift perturbations response due to binding as seen by weighted chemical shift differences (^{1}H and ^{15}N) of glycines ($\Delta\delta$ (ppm) $= [(\delta H)^2 + 0.1 \times (\delta N)^2)]^{1/2}$) between free and bound (saturated with Bax BH3 peptide) Bcl-2 protein in DPC buffer. (*b*) Binding as seen by shift perturbations in ^{1}H and ^{15}N separately plotted as a function of the Bcl-2 residue number. Red and green dotted lines represent the cutoffs for averaged and individual CSP plots, respectively. To see this figure in color, go online.

at the helical BH3-peptide ligands (32). There are also small effects in the TM region containing the C-terminus (G227 to G237) as shown by affected ^{1}H resonances. Except for glycine 5 in the N-terminus region, the other two glycines G8 and G27 near or inherent to neighboring BH4 (10–30) domain show smaller CSPs. As expected, the intrinsically disordered FLD region (33–90 aa) shows no involvement in the binding in this region (G41 to G83), whereas at its connection to the membrane-active BH4 domain of Bcl-2 (33), variations are visible for the glycine residues there.

Glycines as individual Bcl-2 reporters to monitor recognition of the BH3-motif

To obtain a more detailed residue-specific insight into the binding event, we analyzed CSP patterns for individual key glycine residues in different Bcl-2 regions, as seen in Figs. 3 and S8. In general, monitoring changes in the chemical shifts of a protein upon addition of ligands is an ideal method not only to reveal the binding site and the residues involved but also to provide residue-specific information about ligand affinity (27). As seen here, the observed chemical shift patterns and therefore binding features of the Bax-BH3 motif can be categorized into three major distinct groups: 1) peaks that shift linearly upon binding, 2) peaks that deviate from this linearity, and 3) peaks that decrease in intensity or broaden upon binding.

Glycines that follow a linear pattern albeit but display a weak CSP upon binding by retaining sharp and strong NMR resonances (indicating that their relaxation decay is slow) are expected for the flexible loop residues between G36 and G83, as seen in Figs. 2 and S6. Also, these residues show no sign of exchange broadening on the NMR timescale and retain their peak shape.

The observed major peak shift for G5 upon the first titration point followed by very minor further changes indicates a dramatic change in the local environment for the N-terminal part which is linked to the BH4 region. As our

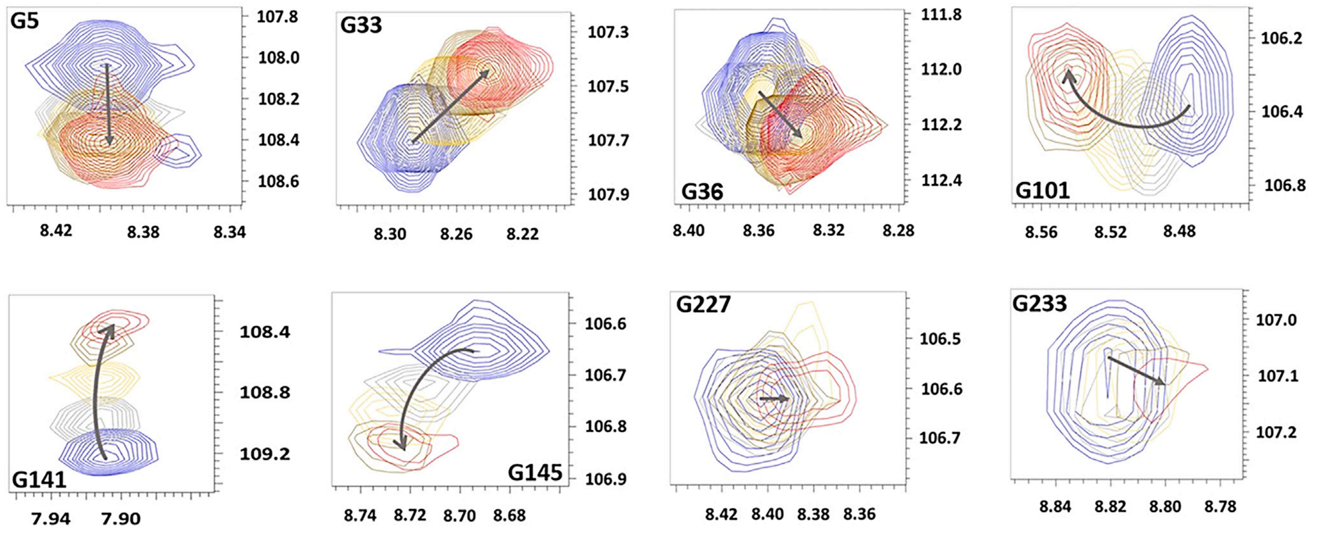

FIGURE 3 Glycine-specific chemical shift responses of Bax-BH3 peptide binding. Different identified glycine residues based on NMR spectra in Fig. S6: residue specific chemical shift perturbations (CSPs) of individual peaks upon increasing addition of the Bax-BH3 peptide (details in legend of Fig. S8). Three different classes of glycine behavior with respect to CSP and peak intensity changes: Class I (G5, G33) high intensity decreasing/strong CSP variation; Class II (G233) low intensity decreasing/weak CSP variations, and Class III: (G141, G203) low intensity decrease/high and complex CSP variations. To see this figure in color, go online.

CLEANEX ^{1}H-^{15}N-TROSY-HSQC experiments (Fig. 1) reveal, G5 and, e.g., G33 are solvent exposed before binding and not embedded in the micelle, which is further supported by slower relaxation decays and overall higher intensities. Interestingly, many of the glycine residues including G33 show a linear behavior of CSP dependency as a function of ligand titration, which possibly indicates a direct (or allosteric) involvement in a single binding mode process (27).

Bcl-2 in its membrane-active state with its BH3-BH1 binding groove region primed for recognizing apoptotic BH3-motifs shows dramatic changes at its glycines in the BH3 and BH1 domains (G101, G141, G145) upon binding of the Bax-BH3 ligand. Besides chemical shift changes as seen in Fig. 2, those glycines show low NMR signal intensities that decrease and broaden significantly upon addition of the BH3-ligand (see also Fig. S8). Those glycines belong to the second major group in Fig. 3 and show a very strong, curved CSP dependence between the ^{1}H and ^{15}N nuclei vector. Those curved CSP effects most likely indicate a combination of a single binding mode and local conformational rearrangements of the Bcl-2 protein itself upon ligand binding (27) as well as structural variabilities in the binding interface region (23). Finally, G223 and G233 in the TM region display a very low intensity in the presence of BH3-ligand exhibiting exchange and a very weak CSP variation.

For most of the identified glycines, we were also able to calculate K_D values, which range from higher μM values to just above 1 mM affinities (Fig. S8 for selected glycines). All those similar K_D values reveal moderate binding affinities across the entire protein. The glycine-specific CSP patterns and varying affinities indicate a simultaneous occurrence of binding that occurs, which nevertheless display a complex behavior, presumably reflecting the domain organization of Bcl-2 and the occurring changes in the membrane-mimicking environment (24). This complexity is also seen for glycines corresponding to the binding domain (G101, G141, G145). Those residues display similar K_D values, but their CSP patterns are different as the respective arrows in Fig. 3 indicate. For G101, the main changes occur at the ^{1}H chemical shift scale, which is indicative for alterations of the chemical environment due to donor/acceptor and/or H-bond changes, both modulating through-space and through-bond interactions (27). For G141 the conclusion is the opposite, with all changes occurring at the ^{15}N vector, with very pronounced variations expected for hydrophobic and polar/charged interactions (27). Here, most residues in this group show an exchange broadening of their NMR resonances due to an increase in the on-off binding events and an exchange rate on the μs-ms timescale upon addition of the BH3-ligand. This type of behavior is seen for glycine residues of the C-terminus, where binding seems to induce slower dynamics. Interestingly, all the different patterns listed here are not randomly located along the sequence, but rather clustered, where different domains seem to display unique binding features.

Complementary, we also used ITC to get an overall macroscopic thermodynamic description of the binding between Bcl-2 and intact, fully functional Bax protein containing its entire BH3-death domain. Although for isolated BH3 domains (not shown), ITC-derived K_D values < mM were observed similar to the NMR-derived values for glycine residues (101, 141, and 145, e.g.), and a K_D of ca. 2 μM was obtained for activated monomeric Bax in DPC micelles (see Fig. S3). Refolded functional Bax has previously been shown to be activated as a monomer in detergent micelles such as octyl glycoside (25,34). For intact, activated Bax, an entropy-driven behavior was seen in the corresponding ITC diagram (Fig. S3), most likely due to the complex micellar environment and entropic changes due to forming a Bax/Bcl-2-containing micelle. Compared with affinity studies on soluble Bcl-2 variants and BH3-ligands where nanomolar values were obtained (ca. 15 nM (19)), the use of a DPC-based micellar system and its complex nature clearly affects the size of the measured affinity, due to additional interactions and unique membrane-mimicking micellar environment, as schematically explained in Fig. S9. In general, proteomicelles (35) have high dissociation constants in the mM range, which means in the case of Bcl-2 the equilibrium between detergent-free and detergent-bound groove binding epitope. Therefore, not surprisingly, our measured K_D values are around 10^3 times lower compared with a detergent-free state, where nM affinities are obtained for soluble truncated versions (23) as well as in vivo cell assays (36). Nevertheless, the trend is clearly the same as found for detergent-free systems in solutions or in membrane-surrounding proteoliposomes. This detergent effect on ligand affinities is quite general and has been seen in many other protein-ligand systems (37,38). Even for intact activated Bax, the affinity to Bcl-2 in detergent was approximately a factor of 30 higher than the value of 35.8 nM as determined by surface plasmon resonance on Bcl-2 stabilized by Brij-35 detergent measured below its critical micelle concentration (39).

Molecular principles for Bcl-2 interaction with apoptotic proteins

Here we used both the Bax-BH3 domain-derived peptide as well as the full-length detergent-activated Bax protein in membrane-mimetics to provide key features of this binding event against its counterplayer, the antiapoptotic Bcl-2 protein. We combined our NMR-derived glycine residue and domain-specific information for Bcl-2 with the overall macroscopic thermodynamic features of the binding process. In activation through the structural rearrangements of Bax where helices undergo rearrangements when they encounter membrane environment, Bax helices 1–9 open up to form the core (helices 1–5), latch (helices 6–8), and TM (helix 9) domains. A schematic picture in Fig. 4 shows the part of the process in the activation of Bax and its

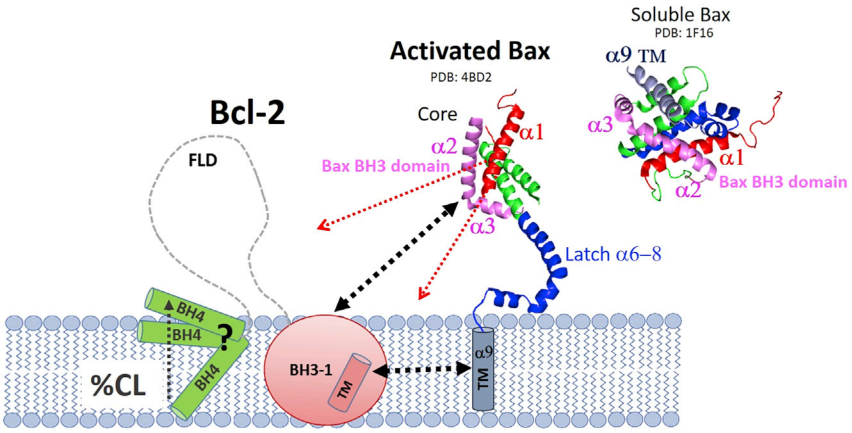

FIGURE 4 Schematic diagram for the binding mechanism between activated Bax protein and membrane-embedded Bcl-2 protein and its involved domains. Soluble Bax (PDB ID: 1F16; (41)) is activated and undergoes rearrangement of helices to membrane-inserted Bax with its distinct TM, latch, and core domains (PDB ID: 4BD2; (42)). Bax BH3 and TM domains are highlighted to interact with dynamically restricted BH1-3 TM domains of Bcl-2 (24,43), whereas the solvent-accessible regulatory flexible loop region (FLD) is flanked outside the membrane surface, as recently observed (12). To see this figure in color, go online.

interaction with Bcl-2 domains. However, even though the complete structural picture still remains elusive, involvement of multiple domains of both Bax and Bcl-2 have been reported in membranes before (40).

Here, several observations are closely associated with using full-length human Bcl-2 in a native-similar membrane environment, which are difficult or impossible to extract using soluble Bcl-2 variants. In most Bcl-2 truncated versions, the TM domain is absent to keep the proteins soluble. Here, the TM part is buried inside the hydrophobic membrane-like core and does not significantly interact with the binding event of the BH3 motif to the Bcl-2 groove region, as the minor changes in Fig. 2 *a* for Gly 227, 233, and 237 indicate. The situation is similar for the extended FLD domain (34–90 aa), where also nearly no changes are observed upon binding as the respective glycines (36–83) in Fig. 1 confirm. Finally, our individual Gly reporters in the hydrophobic groove region of Bcl-2 also indicate a response upon binding where the membrane setting seems to play a modulative role. As the CSP for G101 to G203 in the BH3-BH1-BH3 groove region indicates, there is no common collective response to binding as seen in Fig. 2. As seen for G101 in the BH3 domain and 141 in the BH1 domain, both are heavily affected, whereas BH2 with its G194/193 (overlapping NMR signals) has a more moderate response. This behavior is also supported by severe line broadening of G101 in the BH3 domain and G141/145 in the BH1 domain, as seen in Fig. S7. Finally, glycine residues in the $\alpha2$ to $\alpha8$ helical regions between 97 and 202 aa positions, which connect the BH3-BH1-BH2 domains, show a moderate NMR response (Fig. 2). Most likely, their main role is in ensuring the correct three-dimensional organization of the BH3-BH1-BH2 binding groove in the membrane even under apoptotic membrane-changing conditions (12,15,16).

Looking into the global and local contributions to the binding process in detail via the individual glycine reporters, it is not surprising that the main binding event happens in the BH3-BH1 region of Bcl-2, which forms the extended groove region to recognize—via its individual residues with their varying binding capabilities—BH3-motifs of apoptotic proteins and subsequently to sequester them. As seen by the dramatic changes occurring in the N-terminus and its BH4 domain of Bcl-2 upon Bax-BH3-binding (most pronounced for G5), this domain is sensitive to the membrane environment, and its ligand induced changes as schematically shown in Fig. 4. As seen here, the BH4 domain is separated from the other domains and micelle buried before any binding event, presumably in a quite similar way as in Bcl-x_L where the BH4 domain also was found to be embedded in micelles (30). Presumably, it is important that Bcl-2′s BH4 domain can reside in and out of the mitochondrial membrane and act independently from the main protein core forming the hydrophobic groove region, to regulate many Bcl-2 functions (44) and to prevent activation of Bax and its cell-killing relatives by binding to a novel binding site there (45). Most likely, the presence of cardiolipin in the native MOM membrane determines not only the targeting of Bax to the membrane via its N-terminal $\alpha1$ sequence (46), but it is also highly important in vivo for aligning the BH4 domain in the membrane (11). Not surprisingly the BH4 domain emerged therefore recently as an attractive anticancer target (47) since it is important for the cell-protecting function of Bcl-2 but via its FLD domain and membrane location being independent from the main hydrophobic binding groove formed by BH1-BH3 domains. This Bcl-2 binding groove has been the main drug target in the pharmaceutical industry for many years, with venetoclax being the first licensed drug to interfere with this binding interface to release apoptotic proteins for inducing cancer cell death in various leukemia types (23). Unfortunately, venetoclax is not efficient in many other Bcl-2-sensitive cancers, requiring still major efforts on other Bcl-2 targets like the BH4 domain to combat those tumors in the future.

CONCLUSION

Here, we show the interactions of the BH3 domain of Bax with Bcl-2 in the membrane-mimic environments, using

the backbone glycines of Bcl-2 as probes to monitor the interactions with domains of Bcl-2 and their binding affinities. We also characterize the binding between Bcl-2 and Bax, where proteins are activated in membrane-mimic environments. These studies provide an insight—at a residue-specific resolution—into the individual responses of the functional Bcl-2 protein upon recognizing the key BH3-motifs of apoptotic proteins in membrane-like environments, essential for their sequestration, which is a prime mechanism to tackle cancer by interfering to release cancer cell-killing apoptotic proteins.

SUPPORTING MATERIAL

Supporting Material can be found online at https://doi.org/10.1016/j.bpj.2022.10.041.

AUTHOR CONTRIBUTIONS

A.U.M., J.Å., and G.G. designed the research. A.U.M., K.A., and J.A. performed all experiments. A.U.M. and K.A. analyzed NMR and ITC data. All contributed in generating figures and writing the article.

ACKNOWLEDGMENTS

G.G. acknowledges financial support from the Swedish Research Council, the Swedish Cancer Foundation, the Kempe Foundation, the Knut and Alice Wallenberg Foundation ("NMR for Life" Program), the SciLifeLab, SwedNMR with the Swedish National NMR Center and Umeå Insamlingsstiftelse.

DECLARATION OF INTERESTS

The authors have no competing interests to declare.

REFERENCES

1. Kerr, J. F., A. H. Wyllie, and A. R. Currie. 1972. Apoptosis: a basic biological phenomenon with wide-ranging implications in tissue kinetics. *Br. J. Cancer.* 26:239–257.
2. Cory, S., and J. M. Adams. 2002. The Bcl2 family: regulators of the cellular life-or-death switch. *Nat. Rev. Cancer.* 2:647–656.
3. Westphal, D., G. Dewson, …, R. M. Kluck. 2014. Apoptotic pore formation is associated with in-plane insertion of Bak or Bax central helices into the mitochondrial outer membrane. *Proc. Natl. Acad. Sci. USA.* 111:E4076–E4085.4.
4. Adams, J. M., and S. Cory. 2018. The BCL-2 arbiters of apoptosis and their growing role as cancer targets. *Cell Death Differ.* 25:27–36.
5. Shamas-Din, A., J. Kale, …, D. W. Andrews. 2013. Mechanisms of action of bcl-2 family proteins. *Cold Spring Harb. Perspect. Biol.* 5:a008714.
6. Czabotar, P. E., G. Lessene, …, J. M. Adams. 2014. Control of apoptosis by the BCL-2 protein family: implications for physiology and therapy. *Nat. Rev. Mol. Cell Biol.* 15:49–63.
7. Kale, J., E. J. Osterlund, and D. W. Andrews. 2018. BCL-2 family proteins: changing partners in the dance towards death. *Cell Death Differ.* 25:65–80.
8. Bleicken, S., G. Hofhaus, …, A. J. García-Sáez. 2016. cBid, Bax and Bcl-xL exhibit opposite membrane remodeling activities. *Cell Death Dis.* 7, e2121.
9. Flores-Romero, H., and A. J. García-Sáez. 2019. The incomplete puzzle of the BCL2 proteins. *Cells.* 8:e1176.
10. Lidman, M., Š. Pokorná, …, G. Gröbner. 2016. The oxidized phospholipid PazePC promotes permeabilization of mitochondrial membranes by Bax. *Biochim. Biophys. Acta.* 1858:1288–1297.
11. Vasquez-Montes, V., M. V. Rodnin, …, A. S. Ladokhin. 2021. Lipids modulate the BH3-independent membrane targeting and activation of BAX and Bcl-xL. *Proc. Natl. Acad. Sci. USA.* 118:e2025834118.
12. Mushtaq, A. U., J. Ådén, …, G. Gröbner. 2021. Neutron reflectometry and NMR spectroscopy of full-length Bcl-2 protein reveal its membrane localization and conformation. *Commun. Biol.* 4:507.
13. Campbell, K. J., and S. W. G. Tait. 2018. Targeting BCL-2 regulated apoptosis in cancer. *Open Biol.* 8, 180002.
14. García-Aranda, M., E. Pérez-Ruiz, and M. Redondo. 2018. Bcl-2 inhibition to overcome resistance to chemo- and immunotherapy. *Int. J. Mol. Sci.* 19:3950.
15. Kim, P. K., M. G. Annis, …, D. W. Andrews. 2004. During apoptosis bcl-2 changes membrane topology at both the endoplasmic reticulum and mitochondria. *Mol. Cell.* 14:523–529.
16. Hill, R. B., K. R. MacKenzie, and M. C. Harwig. 2015. The tail-end is only the beginning: NMR study reveals a membrane-bound state of BCL-XL. *J. Mol. Biol.* 427:2257–2261.
17. Schellenberg, B., P. Wang, …, A. P. Gilmore. 2013. Bax exists in a dynamic equilibrium between the cytosol and mitochondria to control apoptotic priming. *Mol. Cell.* 49:959–971.
18. Popgeorgiev, N., L. Jabbour, and G. Gillet. 2018. Subcellular localization and dynamics of the bcl-2 family of proteins. *Front. Cell Dev. Biol.* 6:13.
19. Ku, B., C. Liang, …, B. H. Oh. 2011. Evidence that inhibition of BAX activation by BCL-2 involves its tight and preferential interaction with the BH3 domain of BAX. *Cell Res.* 21:627–641.
20. Ramos, J., J. Muthukumaran, …, T. Santos-Silva. 2019. Shedding light on the interaction of human anti-apoptotic bcl-2 protein with ligands through biophysical and in silico studies. *Int. J. Mol. Sci.* 20:860.
21. Sora, V., and E. Papaleo. 2022. Structural details of BH3 motifs and BH3-mediated interactions: an updated perspective. *Front. Mol. Biosci.* 9, 864874.
22. Petros, A. M., A. Medek, …, S. W. Fesik. 2001. Solution structure of the antiapoptotic protein bcl-2. *Proc. Natl. Acad. Sci. USA.* 98:3012–3017.
23. Birkinshaw, R. W., J. N. Gong, …, P. E. Czabotar. 2019. Structures of BCL-2 in complex with venetoclax reveal the molecular basis of resistance mutations. *Nat. Commun.* 10:2385.
24. Ul Mushtaq, A., J. Ådén, …, G. Gröbner. 2021. Insight into functional membrane proteins by solution NMR: the human bcl-2 protein-A promising cancer drug target. *Molecules.* 26:1467.
25. Ivashyna, O., A. J. García-Sáez, …, P. H. Schlesinger. 2009. Detergent-activated BAX protein is a monomer. *J. Biol. Chem.* 284:23935–23946.
26. Zhu, G., Y. Xia, …, K. H. Sze. 2000. Protein dynamics measurements by TROSY-based NMR experiments. *J. Magn. Reson.* 143:423–426.
27. Williamson, M. P. 2013. Using chemical shift perturbation to characterise ligand binding. *Prog. Nucl. Magn. Reson. Spectrosc.* 73:1–16.
28. Kang, C., N. Bharatham, …, H. S. Yoon. 2012. The natively disordered loop of bcl-2 undergoes phosphorylation-dependent conformational change and interacts with Pin1. *PLoS One.* 7, e52047.
29. Lan, Y. J., P. S. Yeh, …, Y. W. Chiang. 2020. Anti-apoptotic BCL-2 regulation by changes in dynamics of its long unstructured loop. *Commun. Biol.* 3:668.
30. Losonczi, J. A., E. T. Olejniczak, …, S. W. Fesik. 2000. NMR studies of the anti-apoptotic protein Bcl-x(L) in micelles. *Biochemistry.* 39:11024–11033.

31. Denisov, A. Y., G. Chen, …, K. Gehring. 2006. Structural model of the BCL-w-BID peptide complex and its interactions with phospholipid micelles. *Biochemistry.* 45:2250–2256.

32. Garner, T. P., A. Lopez, …, E. Gavathiotis. 2017. Progress in targeting the BCL-2 family of proteins. *Curr. Opin. Chem. Biol.* 39:133–142.

33. Sani, M. A., S. Castano, …, G. Gröbner. 2008. Restriction of lipid motion in membranes triggered by beta-sheet aggregation of the anti-apoptotic BH4 domain. *FEBS J.* 275:561–572.

34. Montessuit, S., G. Mazzei, …, B. Antonsson. 1999. Expression and purification of full-length human Bax alpha. *Protein Expr. Purif.* 15:202–206.

35. Wolfe, A. J., J. F. Gugel, …, L. Movileanu. 2018. Kinetics of membrane protein-detergent interactions depend on protein electrostatics. *J. Phys. Chem. B.* 122:9471–9481.

36. Osterlund, E. J., N. Hirmiz, …, D. W. Andrews. 2022. Efficacy and specificity of inhibitors of BCL-2 family protein interactions assessed by affinity measurements in live cells. *Sci. Adv.* 8:abm7375.

37. Shukla, S., B. Abel, …, S. V. Ambudkar. 2017. Effects of a detergent micelle environment on P-glycoprotein (ABCB1)-ligand interactions. *J. Biol. Chem.* 292:7066–7076.

38. Staus, D. P., L. M. Wingler, …, R. J. Lefkowitz. 2019. Detergent- and phospholipid-based reconstitution systems have differential effects on constitutive activity of G-protein-coupled receptors. *J. Biol. Chem.* 294:13218–13223.

39. Wallgren, M., M. Lidman, …, G. Gröbner. 2013. Reconstitution of the anti-apoptotic bcl-2 protein into lipid membranes and biophysical evidence for its detergent-driven association with the pro-apoptotic Bax protein. *PLoS One.* 8, e61452.

40. Ding, J., Z. Zhang, …, J. Lin. 2010. Bcl-2 and Bax interact via the BH1-3 groove-BH3 motif interface and a novel interface involving the BH4 motif. *J. Biol. Chem.* 285:28749–28763.

41. Suzuki, M., R. J. Youle, and N. Tjandra. 2000. Structure of Bax: coregulation of dimer formation and intracellular localization. *Cell.* 103:645–654.

42. Czabotar, P. E., D. Westphal, …, P. M. Colman. 2013. Bax crystal structures reveal how BH3 domains activate Bax and nucleate its oligomerization to induce apoptosis. *Cell.* 152:519–531.

43. Andreu-Fernandez, V., M. Sancho, …, M. Orzaez. 2017. Bax transmembrane domain interacts with prosurvival Bcl-2 proteins in biological membranes. *Proc. Natl. Acad. Sci. USA.* 114:E1574.

44. Liu, Z., C. Wild, …, J. Zhou. 2016. BH4 domain of Bcl-2 as a novel target for cancer therapy. *Drug Discov. Today.* 21:989–996.

45. Barclay, L. A., T. E. Wales, …, L. D. Walensky. 2015. Inhibition of pro-apoptotic BAX by a noncanonical interaction mechanism. *Mol. Cell.* 57:873–886.

46. Sani, M. A., E. J. Dufourc, and G. Gröbner. 2009. How does the Bax-alpha1 targeting sequence interact with mitochondrial membranes? The role of cardiolipin. *Biochim. Biophys. Acta.* 1788:623–631.

47. Zhang, S. l., R. R. Yang, …, M. Y. Zheng. 2022. Discovery and identification of a novel small molecule BCL-2 inhibitor that binds to the BH4 domain. *Acta Pharmacol. Sin.* https://doi.org/10.1038/s41401-022-00936-0.

Biophysical Journal, Volume *121*

Supplemental information

Domain-specific insight into the recognition of BH3-death motifs by the pro-survival Bcl-2 protein

Ameeq Ul Mushtaq, Jörgen Ådén, Katan Ali, and Gerhard Gröbner

Supplemental information

Domain specific insight into the recognition of BH3-death motifs by the pro-survival Bcl-2 protein

Ameeq Ul Mushtaq, Jörgen Ådén, Katan Ali, and Gerhard Gröbner

Figure S1: **SDS-PAGE of purified, activated Bax protein.**

Figure S2: **Secondary structure determination of refolded, activated Bax protein.**

Figure S3: **ITC experiment of binding intact human Bax protein to Bcl-2 protein.**

Figure S4: **Assignment of Bcl-2 glycine residues**.

Figure S5. Glycine specific insight into Bcl-2 domain dynamics.

Figure S6: **Titration of mBax-BH3 peptide against Bcl-2 protein**.

Figure S7: **$^{1}H^{15}N$ peak intensities of Bcl-2 glycines against mBaxBH3 peptide titration**.

Figure S8: **Glycine residue specific affinities.**

Figure S9: **Schematic picture of the micelle-embedded Bcl-2 and detergent activated monomeric Bax in DPC micelles.**

References

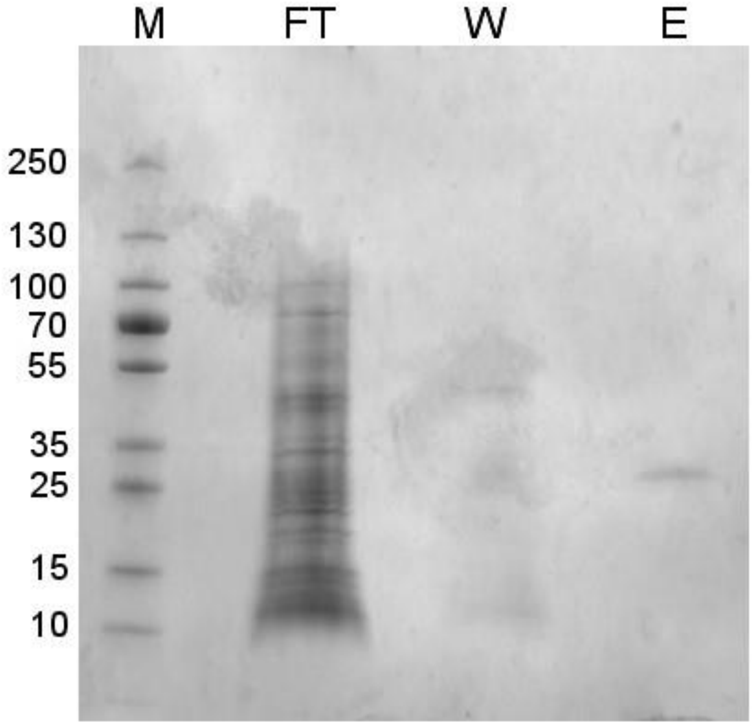

Figure S1. SDS-PAGE of purified, activated Bax protein. Eluted fractions from_nickel affinity purification. Lane M) Molecular weight marker in kDa, FT) Flow-through fraction upon binding refolded, solubilized protein on the column, W) Wash fraction, and E) Eluted, activated Bax protein, recovered from inclusion bodies. Expected molecular weight for Bax with its attached His-tag is 23.3 kDa.

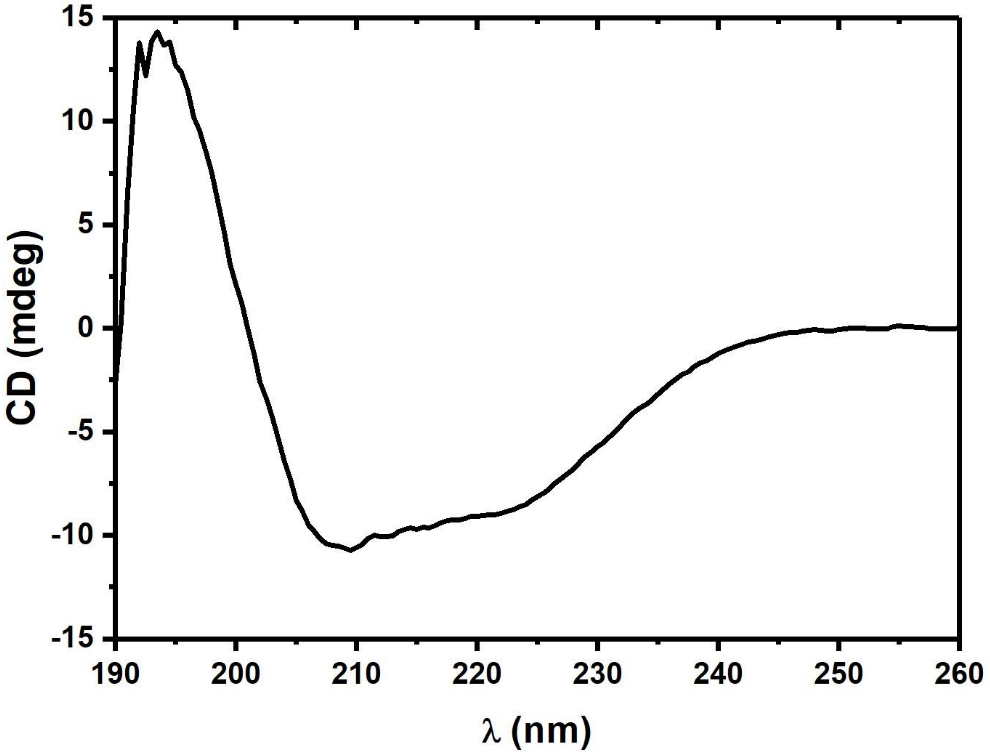

Figure S2. Secondary structure determination of refolded, activated Bax protein. Far-UV CD spectrum of 5 µM activated Bax protein in NMR buffer (20 mM NaPi, 20 mM NaCl, 5 mM DPC, 2 mM TCEP, pH 6.0), recorded at 20 °C. The CD spectrum shows a characteristic helical structure, expected for folded Bax protein.

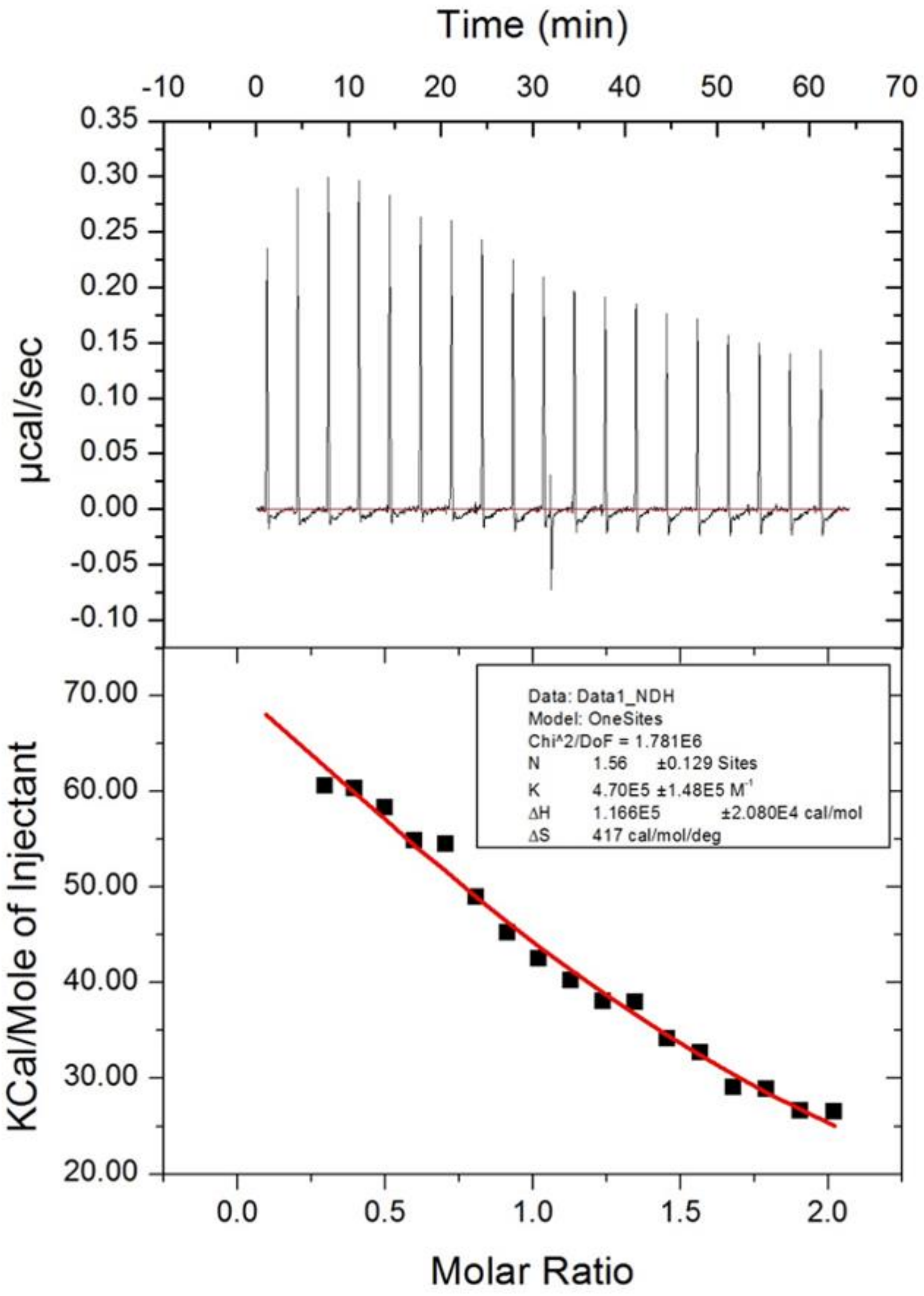

Figure S3. ITC experiment of binding intact human Bax protein to Bcl-2 protein. ITC raw data (top panel) showing differential power (DP) and curve fit (bottom panel) obtained for titrations of 20 µM wild-type human Bax protein into 2 µM intact human Bcl-2 protein. The dissociation constant (K_D) of the binding affinity between both proteins in 5 mM DPC containing NMR buffer was calculated to a K_D value of 2.12 µM. The integrated titration curve (bottom) shows the integrated heat data that was fitted into the one site binding model.

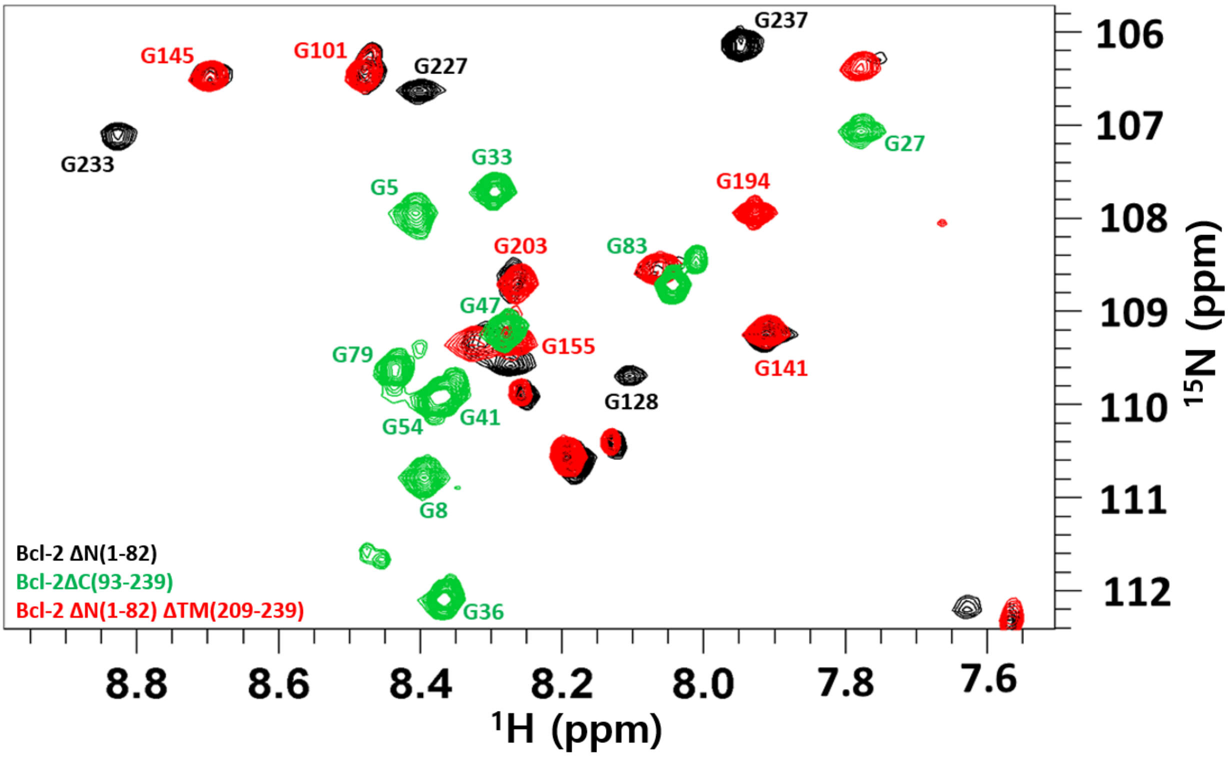

Figure S4. Assignment of Bcl-2 glycine residues. Overlay of the spectral glycine regions of ^{1}H-^{15}N-TROSY-HSQC NMR spectra of Bcl-2 constructs: (black) 0.4 mM ^{15}N-labeled Bcl-2 ΔN(1-82); (green) 0.63 mM ^{15}N-labeled Bcl-2 ΔC(93-239) and (red) 0.25 mM ^{15}N-labeled Bcl-2 ΔN(1-82) ΔC(209-239) protein. All spectra were acquired at 310 K at 850 MHz ^{1}H frequency. Identified glycine residues in the constructs are colored accordingly. Truncation of Bcl-2 protein with fully functional subunits provides less overlapped NMR spectra (s. also [1]).

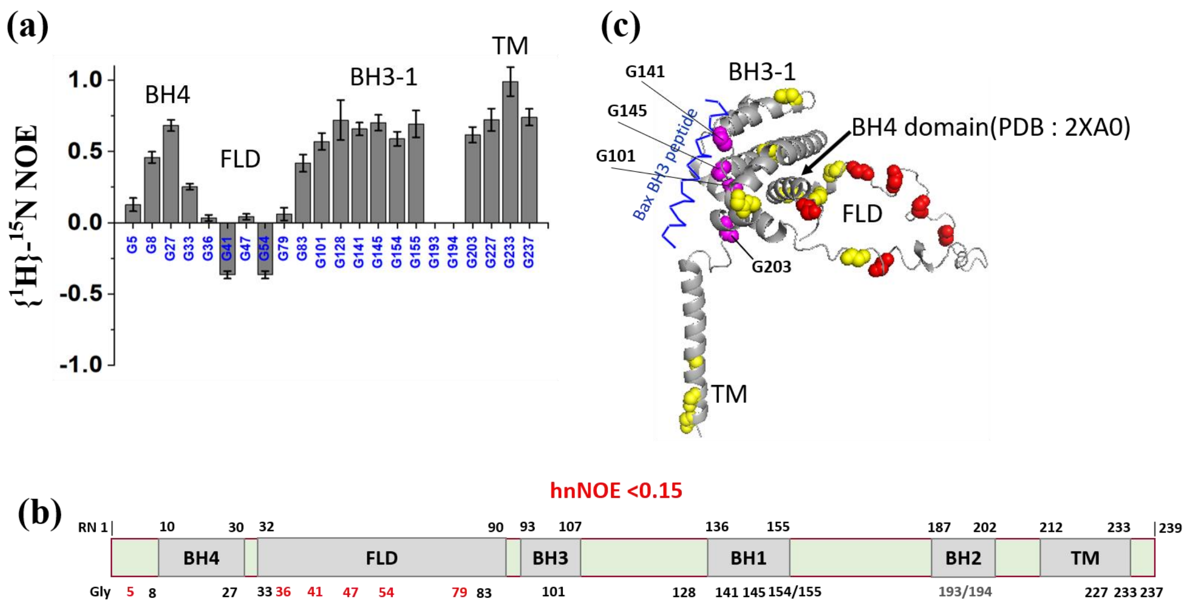

Figure S5. Glycine specific insight into Bcl-2 domain dynamics. (a) Steady-state {^{1}H}-^{15}N heteronuclear NOE plot of the glycine amide groups of Bcl-2 showing ps-ns dynamics of domains in a membrane-mimicking micellar environment. ^{15}N-^{1}H heteronuclear NOE saturation/unsaturation values are plotted on the y-axis between values (−1.0 to 1.0). Errors associated with each NOE value from replicate measurements are shown on top of the bar graphs. **(b)** Glycine residues with hnNOE <0.15 are shown in red along the Bcl-2 sequence. **(c)** Model of the full-length Bcl-2 (where FLD and TM domains are modelled based on the tail-anchored model) derived from truncated Bcl-2 complex (PDB ID: 2XA0). Glycine residues are shown in sphere representations to highlight their distribution in the tail-anchored model, glycines in vicinity to Bax-BH3 peptide are shown in magenta spheres. G141, G145, G101 and G203 are the ones showing major chemical shift perturbations, residues 143-146 i e NWGR of the binding-groove are shown in magenta, glycines' with hnNOE <0.15 are shown in red and the remaining glycines are shown in yellow color spheres.

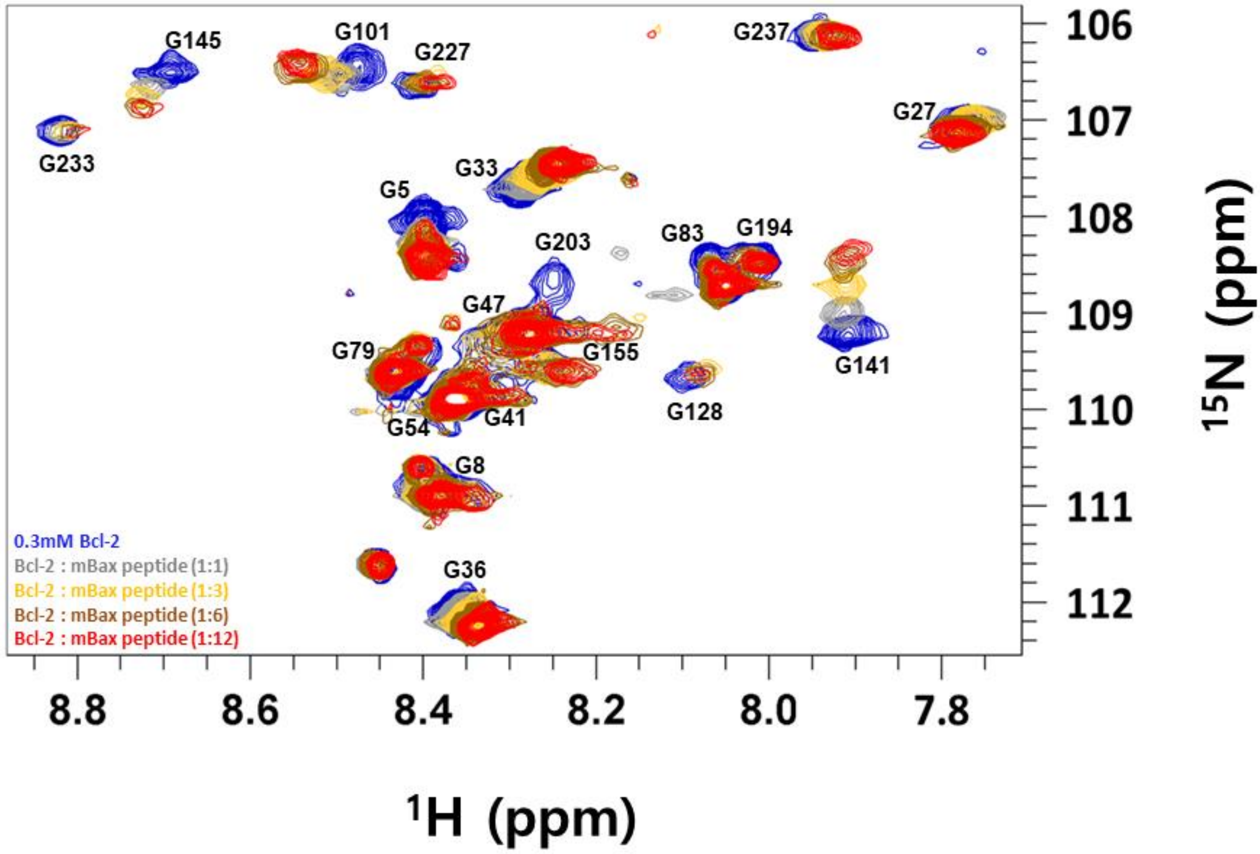

Figure S6. Titration of mBax-BH3 peptide against Bcl-2 protein. Overlay of expanded spectral glycine regions of ^{1}H-^{15}N-TROSY-HSQC NMR spectra showing chemical shift perturbations (CSP's) observed in 0.3 mM ^{15}N-labeled Bcl-2 in NMR buffer upon titration with the Bax-BH3 peptide at various ratios at 310K and at 850 MHz ^{1}H frequency, as described previously [1]. Spectra of Bcl-2 prior (blue) and upon addition of Bax-BH3 peptide added at 1:1, 1:3, 1:6 and 1:12 stoichiometry are indicated in in grey, yellow-orange, brown and red.

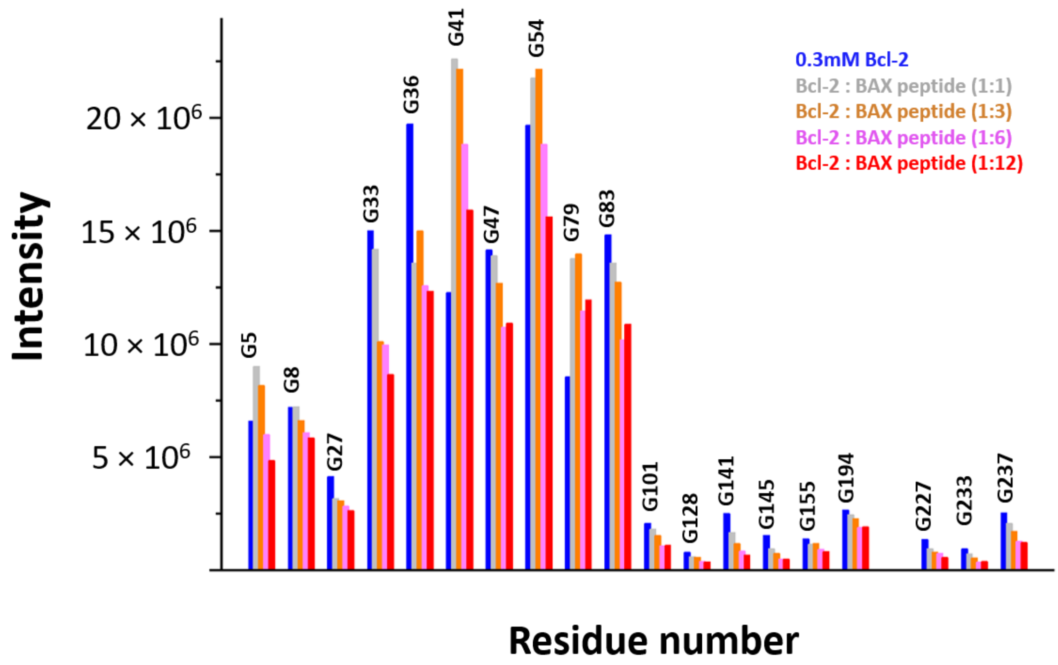

Figure S7. ^{1}H-^{15}N peak intensities of Bcl-2 glycines Bcl-2 as a function of mBax-BH3 peptide concentration. Peak intensities of Bcl-2 glycines against Bax-BH3 peptide at 1:0, 1:1, 1:3, 1:6 and 1:12 protein to peptide molar ratio. Reduced peak intensities of flexible and rigid domains of Bcl-2 along Bax BH3 peptide titration in DPC buffer are plotted.

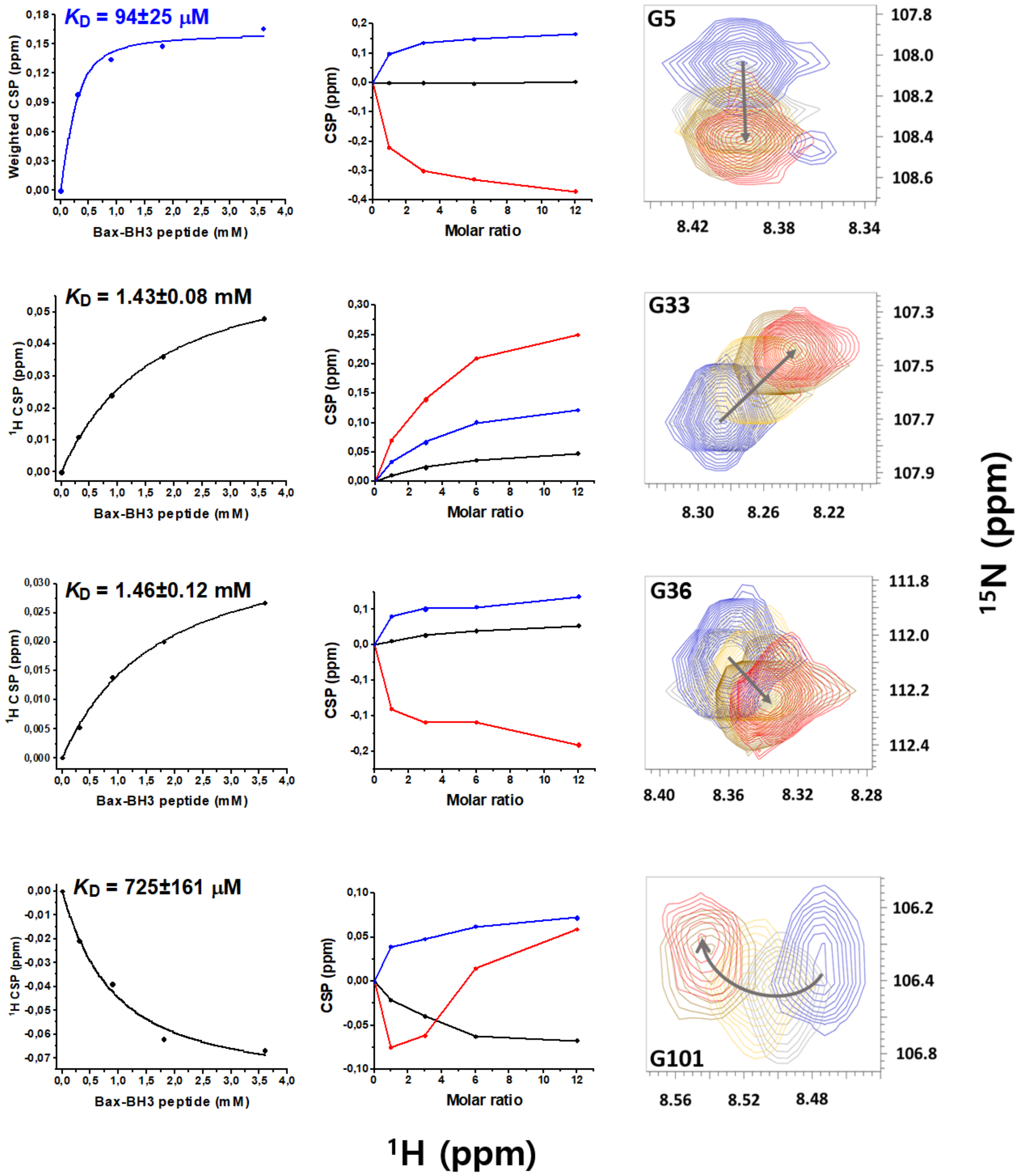

K_D = 94±25 μM
Weighted CSP (ppm)
Bax-BH3 peptide (mM)
CSP (ppm)
Molar ratio
G5
K_D = 1.43±0.08 mM
^1H CSP (ppm)
G33
K_D = 1.46±0.12 mM
G36
K_D = 725±161 μM
G101
^{15}N (ppm)
^1H (ppm)

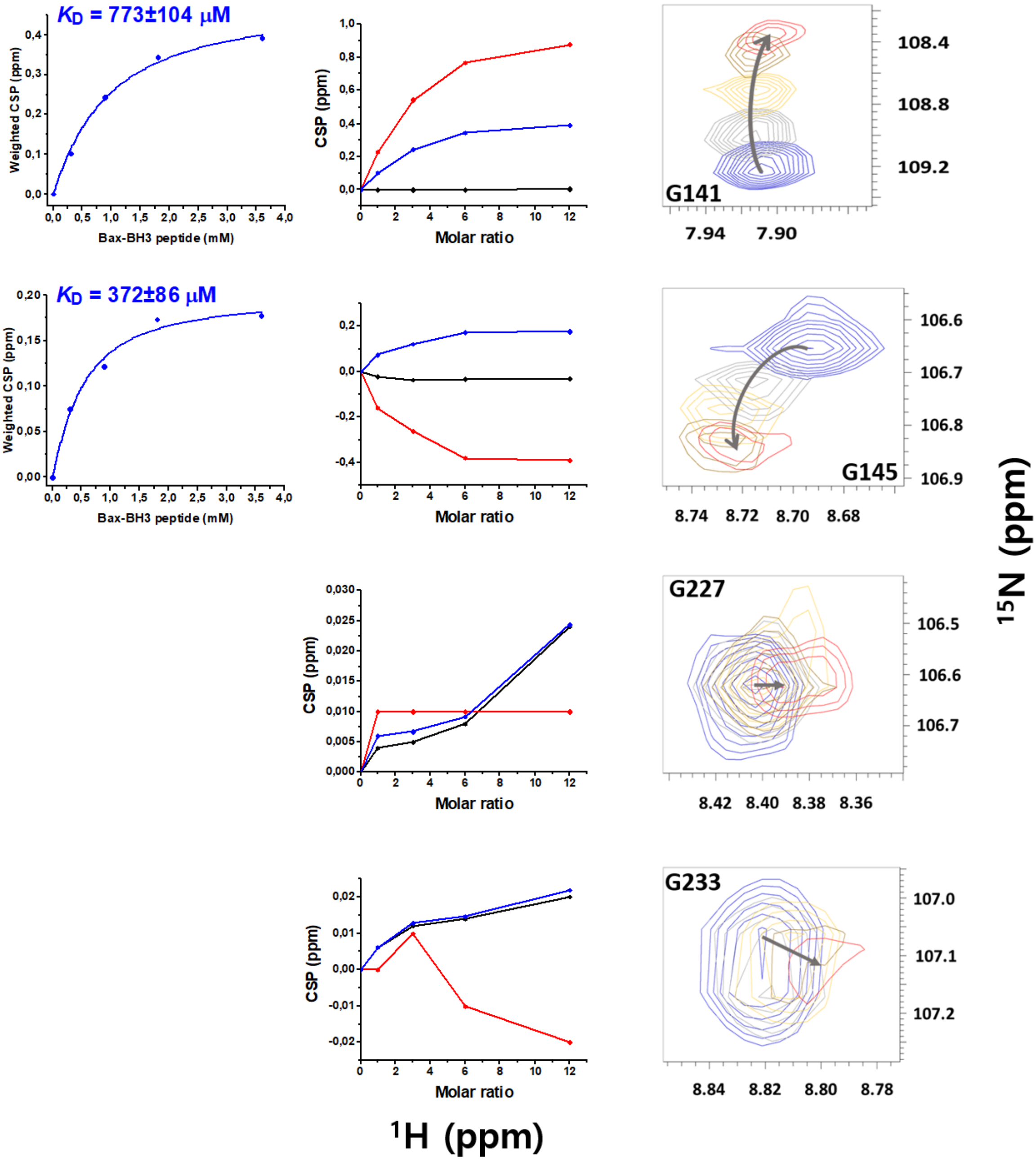

Figure S8. Glycine residue specific affinities. Zoomed boxes (x-axis presents ^{1}H ppm scale; y-axis the ^{15}N ppm scale) show perturbed glycine peaks of the full Bcl-2 spectrum upon addition of the Bax-BH3 peptide. Shown in the middle are the corresponding CSP values for the ^{1}H (black) and ^{15}N (red) nuclei and their weighted average (blue) as described in the Methods section, respectively. For residues showing significant perturbation upon titration (>0.2 ppm), K_D values were calculated and shown (left).

K_D values were calculated either from the ^{1}H, ^{15}N or ^{1}H-^{15}N weighted chemical shifts ($\Delta\delta\ (ppm) = [(\delta H)^2 + 0.1 \times (\delta N)^2)]^{1/2}$) (color coded), based on which fit yielding the lowest chi square value.

K_D values were calculated in OriginPro 2017 (OriginLab Corporation, USA) using the following equation:

$$fb1 = \frac{K_A(E_{tot} + x) + 1}{2 \times K_A \times E_{tot}}$$

$$\sigma = \frac{K_A \times (E_{tot} + x) + 1}{(2 \times K_A \times E_{tot})^2 - x/E_{tot}}$$

$$y = \omega\left(fb1 - \sqrt{(\sigma)}\right)$$

Where E_{tot} is the protein concentration, K_A the association constant, and ω the chemical shift, respectively.

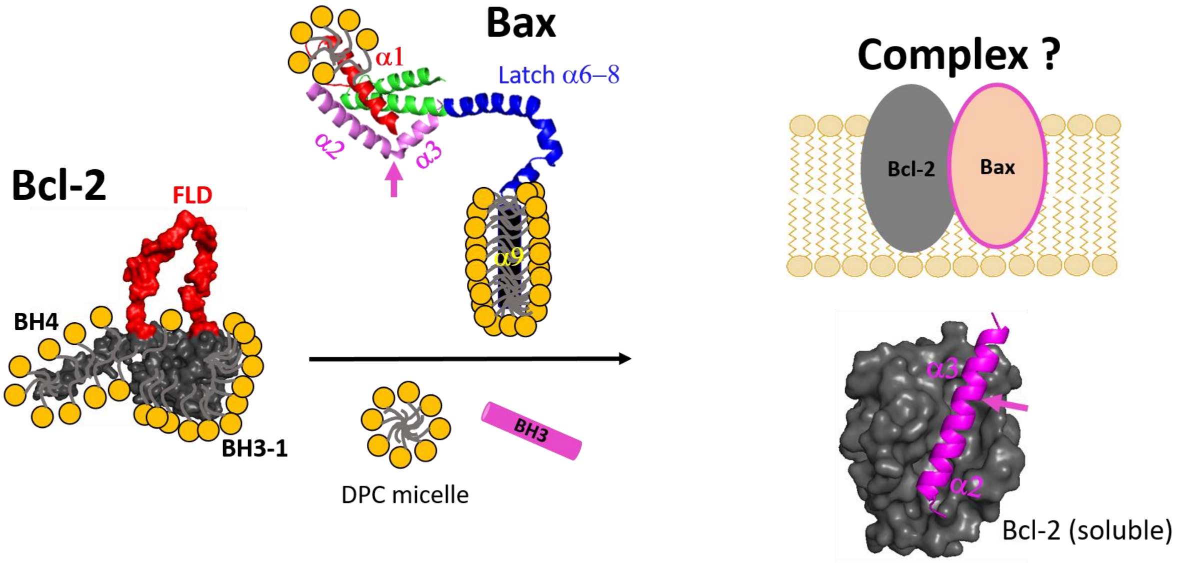

Figure S9. Schematic picture of the micelle-embedded Bcl-2 and detergent activated monomeric Bax in DPC micelles. Detergent-activated monomeric Bax showing exposed BH3 domain (helices 2-3) of the core domain (helices 2-5), latch domain (helices 6-8) and TM (helix 9) domain which is embedded in micelles. Bcl-2 protein in micelles comprises the solvent-accessible regulatory loop domain (FLD), and the micelle embedded BH4 and BH3-1(+TM) domains. Arrows in magenta color show the kink between helices 2 and 3 of activated Bax (PDB ID: 4BD2) which becomes a straight helix in the Bcl-2 bound state (PDB ID: 2XA0).

References

[1] A. Ul Mushtaq, J. Aden, T. Sparrman, M. Hedenstrom, G. Grobner. Insight into Functional Membrane Proteins by Solution NMR: The Human Bcl-2 Protein-A Promising Cancer Drug Target, Molecules 26(5) (2021).
